# GLIS3 drives epithelial–mesenchymal transition and cancer stem–like traits in stomach adenocarcinoma via TGFBR3–Hedgehog signaling

**DOI:** 10.3389/fonc.2026.1826297

**Published:** 2026-05-21

**Authors:** Qi Xiao, Hongyang Deng, Jipin Li, Yijun Zheng, Youcheng Zhang

**Affiliations:** 1 The Second Clinical Medical College of Lanzhou University, Lanzhou, China; 2 Department of General Surgery, The Second Hospital of Lanzhou University, Lanzhou, China

**Keywords:** GLIS3, Hedgehog, EMT, CSC, STAD

## Abstract

**Background:**

GLIS family zinc finger 3 (GLIS3) is a transcription factor implicated in multiple malignancies, but its role in stomach adenocarcinoma (STAD) and its downstream effector axis remain unclear. We investigated whether GLIS3 coordinates epithelial–mesenchymal transition (EMT) and cancer stem cell (CSC)-like programs in STAD through a signaling cascade.

**Methods:**

GLIS3 expression and prognostic associations were analyzed using transcriptomic datasets from The Cancer Genome Atlas, Genotype-Tissue Expression, and Gene Expression Omnibus. GLIS3 protein levels were evaluated by immunohistochemistry in 133 paired STAD tissues. Survival was assessed by Kaplan–Meier analysis together with multivariable regression models. Gain- and loss-of-function studies were performed in AGS and HGC-27 cells to evaluate proliferation, migration, EMT and CSC-like phenotypes. Mechanistic experiments interrogated a GLIS3–transforming growth factor beta receptor 3 (TGFBR3)–Hedgehog axis using ChIP-qPCR, dual-luciferase assays, co-immunoprecipitation, GLI reporter assays, and vismodegib treatment. Tumor growth was assessed in xenograft models.

**Results:**

GLIS3 was consistently upregulated in STAD across public datasets and in the clinical cohort (median H-score: 112 vs 39, *P* < 0.001) and was associated with adverse clinicopathological features and poor survival. In the TCGA cohort, higher GLIS3 expression was associated with greater nodal burden, more advanced nodal and metastatic status, and worse vital status. In multivariable Cox models restricted to cases with defined stage classifications, GLIS3 remained independently associated with worse overall survival in the primary model (hazard ratio = 1.45, 95% confidence interval: 1.04–2.02; *P* = 0.031), with a consistent result in the sensitivity model. Functionally, GLIS3 enhanced proliferation and migration and promoted EMT marker switching and CSC-like traits. Mechanistically, GLIS3 transcriptionally activated TGFBR3 and increased Hedgehog pathway activity; bidirectional rescue experiments showed that TGFBR3 overexpression partially restored, whereas TGFBR3 silencing attenuated, GLIS3-driven EMT/CSC-like phenotypes and growth-related functions. Xenograft experiments supported an *in vivo* growth-promoting role of the GLIS3–TGFBR3–Hedgehog axis.

**Conclusions:**

GLIS3 identifies an aggressive STAD phenotype and engages a TGFBR3–Hedgehog program linked to EMT and CSC-like features. These findings support further evaluation of GLIS3 as a biomarker for risk stratification and provide a rationale for biomarker-guided targeting of the GLIS3–TGFBR3–Hedgehog axis in STAD.

## Introduction

Stomach adenocarcinoma (STAD) is the fourth leading cause of cancer-related deaths worldwide, with both incidence and mortality rates in Asian populations exceeding global averages (1). *Helicobacter pylori* infection, smoking, excessive alcohol consumption, and diet-related exposures remain the primary etiological factors driving STAD (2). Although advances in screening programs and management of *H. pylori* infections have reduced STAD incidence in recent decades, diagnosis often occurs at advanced stages, particularly in regions with limited healthcare access (3). Consequently, the overall prognosis for STAD remains poor, and survival rates for advanced disease remain low (4). Emerging evidence indicates that epithelial–mesenchymal transition (EMT) and enhanced cancer stem cell (CSC) stemness play pivotal roles in tumor progression and therapeutic response (5). While targeted agents have extended survival in some patients with STAD, durable remissions remain rare. These limitations highlight the need to identify actionable molecular drivers that coordinate EMT, CSC plasticity, and immune evasion within the tumor microenvironment, thereby enabling the development of novel precision therapies.

EMT disrupts epithelial polarity and cell–cell adhesion while enhancing cellular motility and invasiveness, enabling carcinoma cells to breach basement membranes, disseminate, and form distant metastases (6). EMT is also closely associated with resistance to chemotherapy, radiotherapy and targeted agents (7). CSCs, including gastric CSCs (GCSCs), exhibit self-renewal capacity and tumor-initiating potential, and survive therapeutic stress through mechanisms such as quiescence, enhanced DNA repair, and drug efflux, thereby contributing to relapse and multidrug resistance (8). Substantial evidence demonstrates that EMT programs and CSC phenotypes are mechanistically linked, jointly maintaining tumor plasticity and heterogeneity (9, 10). Transcription factors that co-regulate EMT and stemness therefore represent promising therapeutic targets for inhibiting invasion–metastasis cascades and overcoming treatment resistance.

GLIS3, a member of the GLIS1–3 subfamily of Krüppel-like zinc finger transcription factors, contains five conserved C2H2 zinc fingers that mediate sequence-specific DNA binding (11). It functions as a transcriptional activator or repressor and is essential for the development and homeostasis of multiple organs, including the pancreas, thyroid and kidneys (12). Germline loss-of-function mutations in GLIS3 cause syndromic neonatal diabetes, congenital hypothyroidism, and polycystic kidney disease, underscoring its pleiotropic physiological roles (13). Aberrant GLIS3 expression or structural rearrangements have been reported in several malignancies, including ependymoma, ductal breast carcinoma, and chromophobe renal cell carcinoma. Notably, the PAX8–GLIS3 fusion is a pathognomonic alteration in hyalinizing trabecular tumors of the thyroid, supporting its oncogenic potential (14). In STAD, GLIS3 has recently been identified as an unfavorable prognostic marker that promotes proliferation, migration, and invasion via the TGF-β1/Smad1/5 pathway (15). However, whether and how GLIS3 directly regulates EMT, sustains cancer stem-like properties, and intersects with downstream signaling pathways in STAD remain unclear. Elucidating these mechanisms is therefore critical for understanding GLIS3-driven tumor progression and for evaluating its potential as a therapeutic target (16).

Given accumulating evidence implicating GLIS3 in epithelial and gastrointestinal malignancies, we hypothesized that GLIS3 promotes STAD progression by enhancing EMT and stem-like traits in tumor cells. In this study, we analyzed GLIS3 expression in STAD versus adjacent normal tissues and assessed its association with clinicopathological features and patient outcomes. Functional experiments demonstrated that GLIS3 enhances both EMT and CSC characteristics, thereby promoting STAD cell proliferation and migration. Mechanistic investigations revealed that this effect is mediated, at least in part, through GLIS3-dependent regulation of its downstream target TGFBR3, which activates Hedgehog signaling to reinforce EMT and stem-like programs. Consistent with these findings, *in vivo* models confirmed the role of the GLIS3–TGFBR3–Hedgehog axis in tumor growth. By delineating this molecular cascade, our study provides new insights into the development of GLIS3-targeted therapeutic strategies in STAD.

## Materials and methods

### Data acquisition and preprocessing

RNA sequencing data for STAD were obtained from The Cancer Genome Atlas (TCGA). Normal gastric tissue data were acquired from the Genotype-Tissue Expression (GTEx) project and integrated with TCGA data for tumor–normal comparisons. External validation datasets were retrieved from the Gene Expression Omnibus (GEO). Gene-level expression values were converted from fragments per kilobase of transcript per million mapped reads (FPKM) to transcripts per million (TPM) using the Toil pipeline, followed by log2(TPM + 1) transformation. When available, paired tumor-adjacent non-tumor samples were prioritized.

### Functional enrichment and EMT/CSC signature analyses

Clinicopathological variables (age, TNM stage, histological grade, etc.) and survival endpoints (overall survival [OS], progression-free survival [PFS], and disease-specific survival [DSS]) were obtained from UCSC Xena. Survival analyzes were conducted using Kaplan–Meier curves and log-rank tests, and hazard ratios with 95% confidence intervals were estimated using Cox proportional-hazards models. For TCGA-based clinicopathological summaries, all annotated cases were retained for descriptive transparency, including those with undefined or non-standard stage-related categories. For Cox proportional-hazards analyzes involving stage-related covariates, however, model-specific restricted datasets were used, and only patients with clearly defined classifications for the variables required by a given model were included. Cases with undefined or discrepant stage-related annotations were excluded from the corresponding regression model.

To explore GLIS3-associated biological programs, GLIS3-correlated genes were identified and subjected to Gene Ontology (GO) and Kyoto Encyclopedia of Genes and Genomes (KEGG) enrichment analysis (17–19). Co-expression networks were visualized using STRING. Gene set enrichment analysis (GSEA) was performed using the Molecular Signatures Database (MSigDB), with genes ranked by their correlation with GLIS3 expression. Gene sets with adjusted *P* < 0.05 and a false discovery rate (FDR) < 0.05 after 1,000 permutations were considered significantly enriched. Relationships between GLIS3 expression, EMT or CSC signatures were examined in bulk RNA-seq data.

### Immunohistochemical staining and evaluation criteria

The STAD tissue cohort used for GLIS3 immunohistochemistry (IHC) comprised paired formalin-fixed, paraffin-embedded (FFPE) tumor and adjacent non-tumor tissues from 133 patients treated at Lanzhou University Second Hospital (Lanzhou, China). Tissues were collected between April 2015 and April 2024. Overall, 147 patients with available paired specimens were identified; 14 patients were lost to follow-up, and 133 patients were ultimately included (106 males and 27 females), with a median age of 67 years (IQR, 62–73). Additional clinicopathological variables, including clinical AJCC stage at diagnosis, grouped pathologic T/N classifications, curative-intent resection, neoadjuvant therapy before sampling, and metastatic-lesion sampling, were retrospectively abstracted from the medical record and pathology reports. Serial sections were deparaffinized and rehydrated according to standard procedures (xylene I/II, 20 min each; graded ethanol). Antigen retrieval was then performed by heat-induced epitope retrieval in citrate buffer (pH 6.0) using a pressure cooker (2.5 min timed after steam release), followed by natural cooling and washing with PBS (pH 7.4). Endogenous peroxidase activity was blocked with 0.3% hydrogen peroxide in methanol for 20 min at room temperature in the dark, followed by PBS washes. Sections were blocked with 5% BSA for 1 h at room temperature. Slides were incubated with an anti-GLIS3 primary antibody (1:200; ab272659; Abcam) overnight at 4 °C in a humidified chamber. The next day, after PBS washes, sections were incubated with a biotinylated secondary antibody provided in an IHC kit (Maixin Biotech, Fuzhou, China; No. KIT-9730; ready-to-use; recognizes mouse/rabbit primary IgG) for 50 min at room temperature. After PBS washes, sections were incubated with streptavidin–peroxidase for 50 min at room temperature. Color was developed using a DAB chromogen (Maixin Biotech; DAB-1031); development time was monitored under a microscope and the reaction was stopped with water. Nuclei were counterstained with Harris hematoxylin for approximately 3 min at room temperature, briefly differentiated for a few seconds, and blued under running tap water. Slides were dehydrated, cleared, and mounted with neutral resin. Microscopic evaluation and image acquisition were performed using a Nikon Ci-S upright microscope (Nikon, Japan). Negative controls were included in each IHC batch by omitting the primary antibody and substituting antibody diluent, with all other steps performed identically to assess nonspecific background staining. An experienced gastrointestinal pathologist, completely blinded to all clinical data, evaluated GLIS3 staining and assigned H-scores. All human tissue studies were approved by the Medical Ethics Committee of Lanzhou University Second Hospital (No. 2024A-960). In accordance with the IRB-approved protocol, eligible participants (or their legally authorized representatives) provided oral informed consent during outpatient visits or remote follow-up for the use of their archived tissue specimens and de-identified clinical data in this study. Participants were informed of data de-identification and confidentiality safeguards, and that refusal or withdrawal would not affect their medical care. All procedures were conducted in accordance with the Declaration of Helsinki.

### Cell culture, gene silencing and overexpression

Human STAD cell lines (MKN-45, AGS, HGC-27, and MKN-7) and the normal gastric epithelial cell line GES-*1* were cultured in RPMI-1640 medium supplemented with 10% fetal bovine serum and 1% penicillin/streptomycin at 37 °C in a humidified incubator with 5% CO_2_. These cell lines were centrally procured by the research center: MKN-45 (IM-H088, Immocell), AGS (IM-H081, Immocell), HGC-27 (IM-H085, Immocell), MKN-7 (CL-0574, Procell), and GES-*1* (IM-H084, Immocell). All cell lines were routinely authenticated by short tandem repeat (STR) profiling and confirmed to be mycoplasma-free. For *in vitro* pathway inhibition, cells were treated with vismodegib (20 μM; HY-10440, MedChemExpress) or an equivalent volume of DMSO as a vehicle control for 48 h.

Total RNA was extracted using TRIzol reagent (R0016, Beyotime), and cDNA was synthesized using a reverse transcription kit (11155ES, Yeasen). Reverse transcription–quantitative real-time PCR (RT-qPCR) was performed using SYBR Green qPCR Mix (1120iES, Yeasen) on a CFX96 Touch Real-Time PCR Detection System (Bio-Rad), with β-actin used as an internal control. Relative expression levels of GLIS3 were quantified using the 2^-^ΔΔCT method (20). Primer sequences were listed in **Supplementary Table 1**.

GES-*1*, MKN-45, AGS, HGC-27, and MKN-7 cells were used for basal GLIS3 expression analyzes by western blotting (WB). Cells were lysed on ice for 30 min in RIPA buffer (P0013B, Beyotime) supplemented with PMSF (ST505, Beyotime). Lysates were clarified by centrifugation at 12,000 × g for 10 min at 4 °C, and supernatants were collected. Protein concentrations were determined using a BCA assay (P0009, Beyotime). Samples were mixed with loading buffer (P0015, Beyotime) and denatured at 95 °C for 10 min. Proteins were separated by SDS-PAGE using 10–12% resolving gels and transferred onto polyvinylidene difluoride (PVDF) membranes (FFP39, Beyotime). Membranes were blocked with 5% non-fat milk at room temperature for 1 h, incubated with primary antibodies overnight at 4 °C, washed three times with TBST for 5 min each, and then incubated with appropriate HRP-conjugated secondary antibodies for 1 h at room temperature. After a final wash, signals were developed using enhanced chemiluminescence (ECL; P0018S, Beyotime) and imaged. Subcellular fractionation was performed to isolate whole-cell lysate, cytosolic, membrane, and nuclear fractions using a commercial fractionation kit (78840, Thermo Fisher Scientific) according to the manufacturer’s protocol.

Primary antibodies included GLIS3 (1:1000, 12678-1-AP, Proteintech), E-cadherin (1:1000, 24E10, CST), N-cadherin (1:1000, 13116, CST), SNAIL (1:2000, abs155342, Absin), CD133 (1:1000, 64326T, CST), EpCAM (1:2000, abs171606, Absin), SOX2 (1:1000, abs115133, Absin), c-Myc (1:1000, 13987, CST), TGFBR3 (1:1000, ab97459, Abcam), GLI1 (1:1000, 2534, CST), PTCH1 (1:500, 17520-1-AP, Proteintech), HHIP (1:1000, ab230271, Abcam), SUFU (1:1000, 2520, CST), Na^+^/K^+^-ATPase (1:5000, 55187-1-AP, Proteintech), Lamin B1 (1:5000, 12987-1-AP, Proteintech), MKI67 (1:500, ab92742, Abcam), β-actin (1:8000, 20536-1-AP, Proteintech), and GAPDH (1:8000, abs132004, Absin). Secondary antibodies included SA00001–1 and SA00001-2 (1:5000, Proteintech). Antibody specificity was supported by manufacturer-provided validation data, including IHC-validated antibodies, and signal intensities within the linear range were proportional to protein loading.

For transient knockdown experiments, cells were seeded in six-well plates and cultured to 50–60% confluency before transfection with Lipofectamine 3000 (L3000015, Thermo Fisher Scientific) according to the manufacturer’s protocol. Two independent siRNA sequences were designed for GLIS3 and TGFBR3, together with the corresponding negative-control siRNAs, and were provided by GeneChem. After 48–72 hours of incubation, cells were collected for subsequent analysis, including RT-qPCR and WB, to verify transfection efficiency.

For overexpression experiments, cells were transfected with overexpression plasmids. The GLIS3 overexpression plasmid (OE-GLIS3; pcDNA3.1(+)-GLIS3), the TGFBR3 overexpression plasmid (OE-TGFBR3; pcDNA3.1(+)-TGFBR3), and the corresponding empty vector control (pcDNA3.1(+)) were synthesized and provided by GeneChem (Shanghai, China). Cells were transfected at 60–70% confluency, using Lipofectamine 3000 (L3000015, Thermo Fisher Scientific) according to the manufacturer’s instructions. Cells were incubated for 48–72 hours, and overexpression efficiency was confirmed by RT-qPCR and WB.

For stable knockdown experiments, cells were cultured to 40–50% confluency before infection with lentiviral particles provided by GeneChem, with Polybrene (C0351, Beyotime) added at a final concentration of 5 μg/mL to enhance transduction efficiency. The shRNA cassette was cloned into the lentiviral shRNA vector GV248 (GeneChem). After 48 h, puromycin (2 μg/mL) was applied for continuous selection of stable knockdown clones, which were further validated by RT-qPCR and WB analysis. Knockdown experiments were performed in AGS and HGC-27 cells, and overexpression experiments were conducted in AGS and HGC-27 cells. For rescue experiments, AGS cells were assigned to si-NC + Vector, si-GLIS3 + Vector, si-NC + OE-TGFBR3, and si-GLIS3 + OE-TGFBR3 groups, whereas HGC-27 cells were assigned to Vector + si-NC, OE-GLIS3 + si-NC, Vector + si-TGFBR3, and OE-GLIS3 + si-TGFBR3 groups. Target genes included *GLIS3* and *TGFBR3* for both knockdown and overexpression. All relevant sequences are listed in **Supplementary Table 1**.

### Functional and mechanistic assays

For wound-healing assays, cell migration was assessed by creating a wound in confluent cell monolayers using a sterile pipette tip. Cells were cultured in serum-free medium, and images were captured at 0, 24, and 48 hours.

Cell proliferation was assessed using the Cell Counting Kit-8 (CCK-8) assay. Cells were seeded in 96-well plates, and proliferation was measured every 24 h using the CCK-8 reagent. Absorbance was measured at 450 nm to quantify cell proliferation over a 4-day period.

For colony-formation assays, cells were seeded at a density of 1 × 10^3^ cells per well in six-well plates and cultured for 10–14 days. For cells subjected to transient transfection, re-transfection was performed every 4 days during the 14-day period. After incubation, cells were fixed with formaldehyde and stained with crystal violet, followed by imaging and documentation. AGS and HGC-27 cells were used for migration and proliferation experiments. Rescue experiments were further evaluated by wound-healing, CCK-8, and colony-formation assays using the grouping strategies described above.

Chromatin immunoprecipitation (ChIP) assays were performed using a ChIP Assay Kit (P2078, Beyotime). Briefly, approximately 1 × 10^6^ cross-linked cells were collected per tube. After the addition of 200 μL of cell lysis buffer, cells were lysed for 15 min and centrifuged at 1000 × g for 5 min at 4 °C. The lysate was sonicated to shear DNA into fragments of 200–1000 bp. Antibodies against GLIS3 (169792-MSM1-P0, Thermo Fisher Scientific), TGFBR3 (2519, CST) and IgG (rabbit: 2729, CST; mouse: 5415, CST) were used for immunoprecipitation. DNA was purified using a DNA Purification Kit (D0033, Beyotime) and quantified by qPCR. ChIP–qPCR assays were performed in AGS and HGC-27 cells to assess GLIS3 occupancy at the TGFBR3 promoter. In addition, ChIP–qPCR was used to quantify crosslinking-dependent enrichment of predefined GLI1 promoter amplicons recovered with an anti-TGFBR3 antibody (**Supplementary Figure 1C**); these data are reported as an exploratory observation and were not used to infer direct TGFBR3–DNA binding.

Co-immunoprecipitation (Co-IP) assays were performed using an Immunoprecipitation Kit (P2179S, Beyotime) according to the manufacturer’s protocol. AGS and HGC-27 cells were lysed in RIPA buffer, and lysates were clarified by centrifugation at 12,000 × g for 10 min at 4 °C. Precleared lysates were incubated overnight at 4 °C with rotation using primary antibodies against GLIS3 (1:50, 169792-MSM1-P0, Thermo Fisher Scientific), TGFBR3 (1:100, 2519, CST), GLI1 (1:50, 2534, CST), or SUFU (1:50, 2522, CST), along with species-/isotype-matched IgG controls (equal amount, rabbit: 2729, CST; mouse: 5415, CST), to allow immunoprecipitation. The following day, protein A/G agarose beads were added to capture antibody–protein complexes. After elution, samples were resolved by SDS-PAGE, transferred to PVDF membranes, and probed with primary and secondary antibodies. Co-IP assays were conducted in both AGS and HGC-27 cells.

For dual-luciferase assays, 1 × 10^4^ cells were seeded per well in 96-well plates and cultured for 24 h. Cells were then co-transfected with luciferase reporter plasmids and designated siRNAs. After 5 h of incubation in serum-free medium, the transfection mixture was replaced with complete medium. Following an additional 48 h of culture, cells were lysed, and firefly and Renilla luciferase activities were measured sequentially. Assays were performed in AGS and HGC-27 cells to evaluate the regulatory relationship between GLIS3 and the TGFBR3 promoter. Two putative GLIS3-responsive motifs within the TGFBR3 promoter were identified by Tsingke (Mut 1: GGGCGG-GTTAAA; Mut 2: CCTGGA-CAATTC). Wild-type and corresponding mutant promoter fragments were cloned into luciferase reporter vectors. Mutations were designed to disrupt the predicted motifs without substantially altering the overall promoter context. The 8× GLI-responsive luciferase reporter plasmid used in the assays was synthesized by Tsingke Biotechnology.

### Xenograft model

To minimize fight-related stress and variability in subcutaneous xenografts, female BALB/c-nu mice (SPF grade; 15–20 g, 6–8 weeks old; supplier: BestCell, No. 4220235000007747) were used. Animals were housed in a barrier facility under controlled conditions (22 ± 2 °C, 45–65% relative humidity, 12 h light/dark cycle) with ad libitum access to food and water, with three to five mice per cage. Mice were randomly allocated into three groups (n = 5 per group; total n = 15). Anesthesia for tumor cell implantation was induced by intraperitoneal injection of sodium pentobarbital (3% w/v, P3761, Sigma) at a dose of 50 mg/kg, and anesthetic depth was confirmed by loss of the toe-pinch reflex. Because the procedure was minimally invasive, no analgesics were administered. Each mouse received a single subcutaneous injection of 5 × 10^6^ HGC-27 cells into the right axilla (vector control, vismodegib treatment, and stable GLIS3 knockdown groups). When xenografts reached approximately 100–150 mm^3^, vismodegib was administered by oral gavage at 100 mg/kg once daily until the end of the experiment.

Animal health and behavior were monitored at least once daily, including general condition, food and water intake, and activity. Post-anesthesia recovery was observed until normal ambulation resumed. Tumor volume was measured every 3–4 days using calipers and calculated as V = (L × W^2^)/2, and body weight was recorded at the same intervals. Welfare measures included stable co-housing, environmental enrichment, appropriate anesthesia, and monitored recovery. Humane endpoints were predefined as follows: (1) tumor volume ≥ 1,500 mm^3^ or evidence of ulceration, necrosis, or self-mutilation; (2) body-weight loss > 15% from baseline or > 10% over 2 consecutive days; (3) persistent lethargy, marked impairment of locomotion or feeding, respiratory distress, or inability to access food/water; or (4) any moderate-to-severe pain not amenable to relief. At 21 days after tumor implantation, or earlier upon reaching a humane endpoint, mice were euthanized by intraperitoneal overdose of sodium pentobarbital (200 mg/kg). Death was confirmed by cessation of spontaneous respiration and heartbeat, loss of the corneal reflex, and absence of response to noxious stimuli. All 15 mice completed the study and were euthanized according to protocol, with no unanticipated deaths observed. Excised xenograft tissues were divided for molecular and histologic analyzes; paraffin-embedded tumor sections were additionally subjected to immunohistochemical staining for TGFBR3, GLI1, and E-cadherin using the procedures described above for paraffin sections. All animal procedures complied with institutional guidelines for the care and use of laboratory animals and were approved by the Ethics Committee of the Second Clinical Medical College of Lanzhou University (No. D2024-691).

### Statistical analysis

All statistical analyzes were performed using R software (v4.2.3) and GraphPad Prism (v9.5). Continuous variables are presented as median (interquartile range, IQR) and were compared using the Wilcoxon rank-sum test. Categorical variables were compared using the χ² test or Fisher’s exact test, as appropriate. Correlations between continuous variables were evaluated using Pearson or Spearman correlation coefficients, as appropriate. Samples were dichotomized into high- and low-expression groups using the median GLIS3 expression as the cutoff. Survival curves were generated using the Kaplan–Meier method and compared using the log-rank test. In the TCGA cohort, hazard ratios (HRs) and 95% confidence intervals (CIs) were estimated using Cox proportional-hazards models. For descriptive clinicopathological analyzes, percentages for categorical variables were calculated using the total number of patients in each GLIS3 group as the denominator, and unknown categories were retained for transparency. For TCGA multivariable Cox analyzes involving stage-related covariates, only cases with clearly defined classifications for the variables included in a given model were analyzed. The primary TCGA multivariable model included GLIS3 expression, age, sex, and grouped AJCC pathologic stage (I/II vs III/IV); a sensitivity analysis used four-level AJCC pathologic stage with Stage I as the reference category. Pathologic M stage was assessed in univariable analysis but was not entered into multivariable models containing AJCC pathologic stage because M status is a component of AJCC stage. In the local IHC cohort, overall survival in the curative-intent resection, primary-tumor subset was further evaluated using Weibull accelerated failure time models. To reduce overfitting and avoid within-model collinearity among overlapping stage-related variables, two parsimonious multivariable models were used: a primary model including GLIS3 expression and clinical AJCC stage at diagnosis, and a sensitivity model including GLIS3 expression and pathologic N stage. Multi-group comparisons were performed using one-way or two-way ANOVA, as appropriate. Two-way ANOVA was used to assess the main effects of si-TGFBR3 and vismodegib and their interaction. Unless otherwise specified, all tests were two-sided. Statistical significance was set at *P* < 0.05, and results were denoted as *P < 0.05, ** *P* < 0.01, and *** *P* < 0.001; ns denotes not significant (*P* ≥ 0.05).

## Results

### GLIS3 is upregulated in STAD and associated with poor prognosis

**Figure 1:**
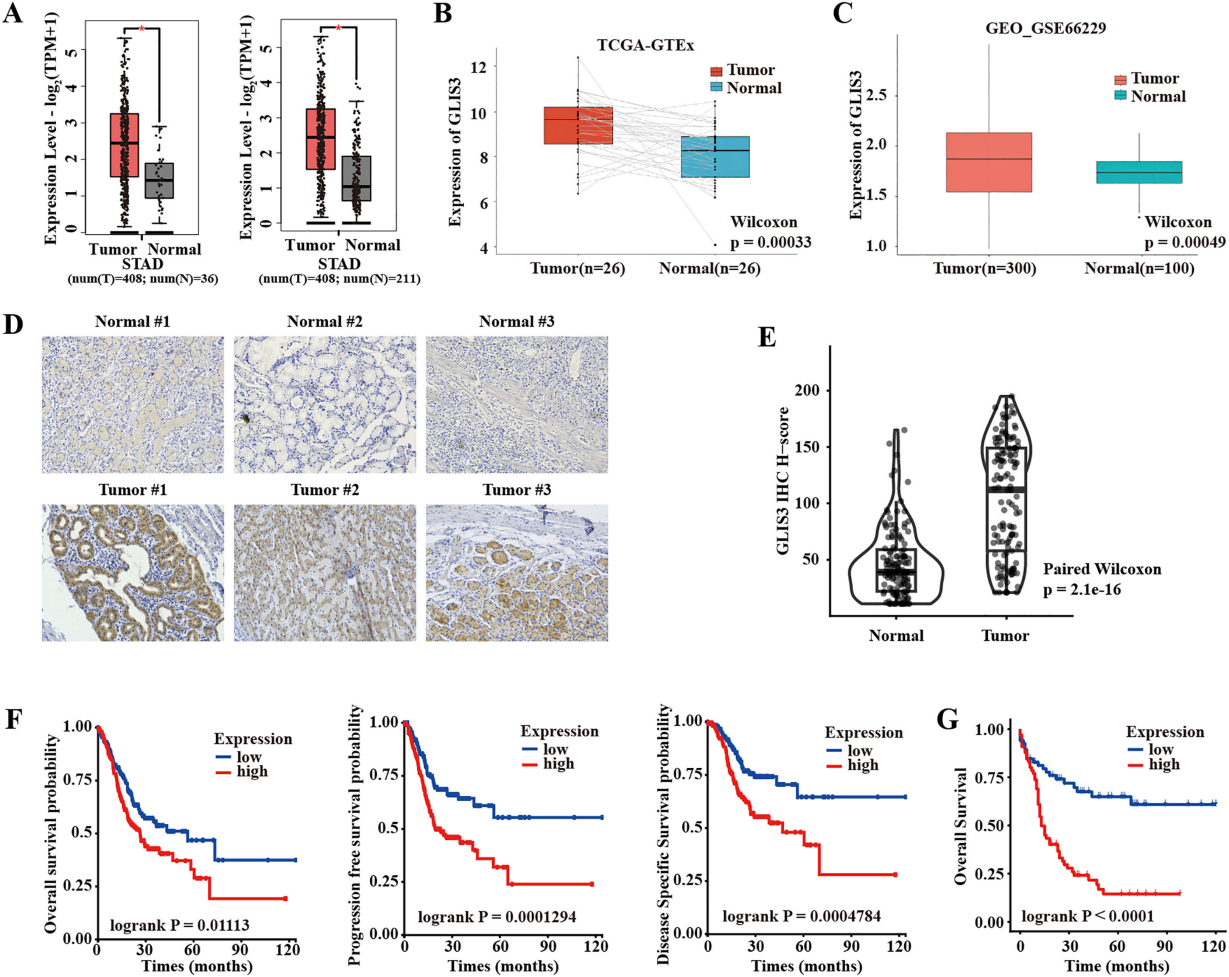
Elevated GLIS3 expression in STAD and its association with adverse clinicopathological features and prognosis. **(A)** Differential GLIS3 mRNA expression between STAD tissues and normal gastric tissues based on TCGA-STAD and combined TCGA–GTEx datasets; **(B)** Paired comparison of GLIS3 mRNA levels in TCGA-STAD tumor samples and their matched adjacent non-tumor tissues; **(C)** External validation of GLIS3 overexpression in STAD using the GEO dataset GSE66229; **(D)** Paired IHC analysis of GLIS3 expression in tumor and adjacent normal tissues from 133 STAD patients; **(E)** IHC H-score of GLIS3 expression in tumor and adjacent normal tissues; **(F)** K–M survival curves showing the association between GLIS3 expression and OS, PFS, and DSS in the TCGA-STAD cohort; **(G)** K–M analysis of OS according to GLIS3 expression levels in the IHC cohort. * *P* < 0.05, ** *P* < 0.01, and *** *P* < 0.001; ns, *P* ≥ 0.05; IHC, Immunohistochemistry; K–M, Kaplan–Meier; OS, Overall survival; PFS, Progression-free survival; DSS, Disease-specific survival.

Analysis of the TCGA-STAD cohort revealed that GLIS3 mRNA levels were significantly higher in tumor tissues than in adjacent normal tissues, and this differential expression was further confirmed when TCGA data were integrated with GTEx normal gastric samples (*P* < 0.05; **Figure 1A**). In a paired analysis of TCGA samples, GLIS3 expression remained markedly elevated in tumors compared with matched non-tumor tissues (*P* < 0.001; **Figure 1B**). External validation using the GEO dataset GSE66229 yielded consistent results, demonstrating robust GLIS3 overexpression in STAD (*P* < 0.001; **Figure 1C**). In our IHC cohort of 133 paired cases (**Figure 1D**), the median H-score for GLIS3 was 39 in adjacent normal tissue versus 112 in tumor tissue (*P* < 0.001; **Figure 1E**). In the TCGA cohort, higher GLIS3 expression was associated with greater positive lymph node burden, more advanced grouped pathologic N status, more frequent metastatic disease, more advanced grouped AJCC pathologic stage, and poorer vital status (**Supplementary Table 2**). Kaplan–Meier analysis further showed that patients in the high-GLIS3 group had significantly shorter OS (*P* < 0.05), PFS (*P* < 0.001), and DSS (*P* < 0.001) (**Figure 1F**). In model-specific restricted multivariable analyzes, GLIS3 remained independently associated with worse OS across the primary stage-based model and the sensitivity model (**Supplementary Table 3**). In the independent IHC cohort, higher GLIS3 H-scores were associated with more advanced clinical AJCC stage at diagnosis, T3/T4 disease, and N2/N3 nodal status (**Supplementary Table 4**). Consistently, patients with high GLIS3 expression exhibited markedly shorter OS compared with those with low expression (*P* < 0.001; **Figure 1G**). In supplementary survival analyzes restricted to the curative-intent resection, primary-tumor subset, high GLIS3 expression remained significantly associated with shorter OS after adjustment for either clinical AJCC stage at diagnosis in the primary model or pathologic N stage in the sensitivity model (**Supplementary Table 5**). Taken together, these findings indicate that elevated GLIS3 expression is associated with more aggressive disease features and poorer clinical outcomes in STAD.

### GLIS3-associated transcriptional programs are enriched for EMT and stemness signatures

**Figure 2:**
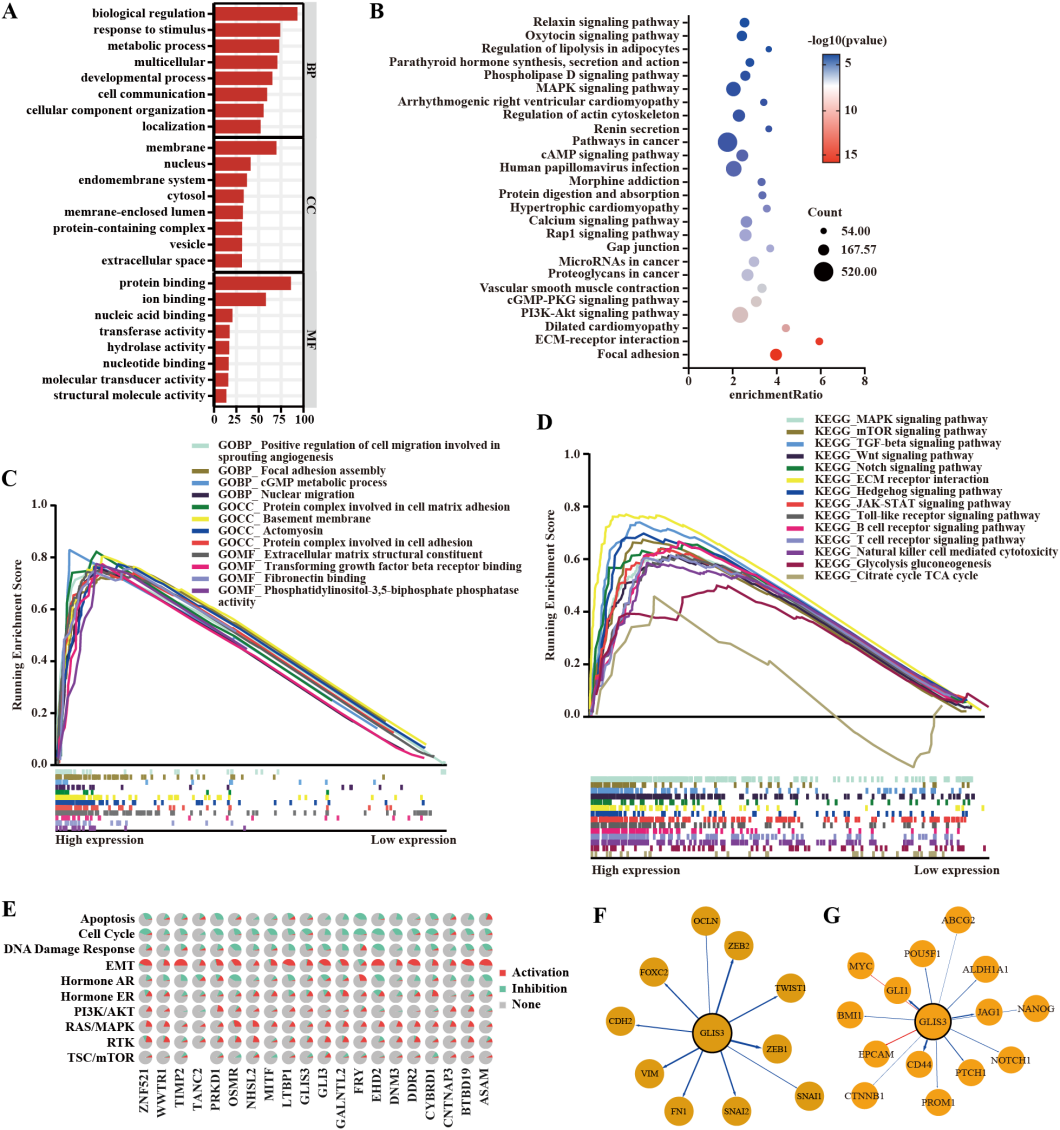
GLIS3 enrichment analysis and its association with EMT and CSCs. **(A)** GO enrichment analysis of genes co-expressed with GLIS3; **(B)** KEGG pathway enrichment analysis of genes co-expressed with GLIS3; **(C)** GSEA of GO functional categories; **(D)** GSEA of KEGG pathway enrichment; **(E)** Functional enrichment analysis of GLIS3-related genes; **(F)** Correlation between GLIS3 expression and EMT-related genes; **(G)** Correlation between GLIS3 expression and CSC-related genes. EMT, Epithelial–mesenchymal transition; CSCs, cancer stem cells; GO, Gene Ontology; KEGG, Kyoto Encyclopedia of Genes and Genomes; GSEA, Gene set enrichment analysis.

GO and KEGG enrichment analyzes of GLIS3-positively correlated genes revealed enrichment in key biological processes, including biological regulation, metabolic processes, developmental pathways, and protein binding (**Figure 2A**). KEGG pathway analysis further highlighted the involvement of GLIS3-associated genes in tumor-related signaling pathways, including the MAPK pathway (**Figure 2B**). GSEA indicated that elevated GLIS3 expression was associated with positive regulation of cellular migration and angiogenesis (**Figure 2C**). Additional pathway-based analyzes underscored significant associations with the MAPK, Wnt, and Hedgehog signaling pathways (**Figure 2D**). These molecular functions and signaling cascades are critically involved in STAD progression and are closely linked to EMT and CSC characteristics.

Subsequent pathway activity analysis of the top 20 GLIS3-associated genes demonstrated a strong correlation with EMT-related features (**Figure 2E**). To further delineate the relationship between GLIS3, EMT, and CSC phenotypes, we analyzed the expression of EMT-related genes in relation to GLIS3 levels (**Figure 2F**). Similarly, associations between GLIS3 and CSC-related genes were examined (**Figure 2G**). Collectively, these findings suggest that GLIS3 may influence EMT and cancer stem-like transcriptional programs, prompting further functional investigation of its role in regulating proliferation, migration, and stemness in STAD cells.

### GLIS3 promotes proliferation, migration, EMT and stem-like traits in STAD cells

**Figure 3:**
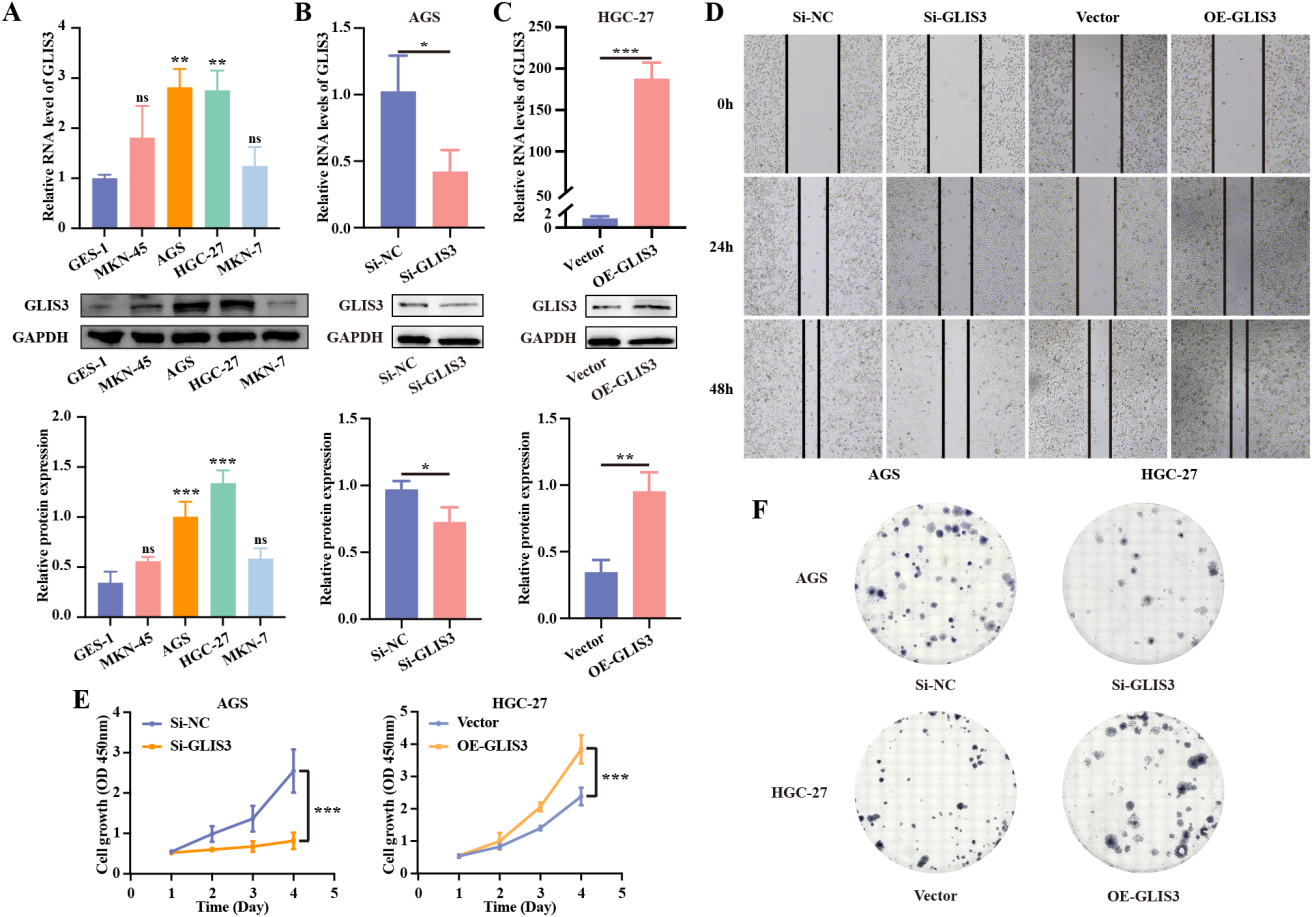
GLIS3 promotes proliferation, migration, EMT and CSC-like traits in STAD cells. **(A)** Basal mRNA and protein expression levels of GLIS3 in the normal gastric epithelial cell line GES-*1* and four gastric cancer cell lines (MKN-45, AGS, HGC-27, and MKN-7); **(B)** Verification of GLIS3 knockdown efficiency in AGS cells by RT-qPCR and WB; **(C)** Verification of GLIS3 overexpression efficiency in HGC-27 cells by RT-qPCR and WB (OE-GLIS3 insert corresponds to NM_001042413.2 nt 747–3539); **(D)** Wound-healing assays demonstrating the effects of GLIS3 knockdown and overexpression on cell migration; **(E)** CCK-8 assays assessing the impact of GLIS3 on cell proliferation; **(F)** Colony formation assays evaluating clonogenic growth following GLIS3 knockdown or overexpression. * *P* < 0.05, ** *P* < 0.01, and *** *P* < 0.001; ns, *P* ≥ 0.05 (n = 3 independent experiments); RT-qPCR, Reverse transcription–quantitative real-time PCR; WB, Western blotting.

To further clarify the functional role of GLIS3 in STAD cells, we performed a series of *in vitro* assays. RT-qPCR and WB analyzes across four gastric cancer cell lines showed an overall pattern of higher GLIS3 expression relative to the normal gastric epithelial cell line GES-1, although the magnitude of increase varied among cell lines (**Figure 3A**). Transient GLIS3 knockdown was achieved by siRNA transfection, whereas GLIS3 overexpression was induced by plasmid transfection. In the principal experiments, GLIS3 knockdown in AGS cells and GLIS3 overexpression in HGC-27 cells were verified by RT-qPCR and WB (*P* < 0.05; **Figures 3B, C**). As complementary validation, GLIS3 overexpression in AGS cells and GLIS3 knockdown in HGC-27 cells were likewise confirmed at both the mRNA and protein levels (**Supplementary Figures 3A, B**). To further address sequence-specificity concerns, two independent siRNAs targeting GLIS3 were screened in AGS cells, and both reduced GLIS3 expression at the mRNA and protein levels; the sequence with the stronger and more reproducible knockdown was used in the subsequent loss-of-function experiments (**Supplementary Figure 3C**). Wound-healing assays demonstrated that GLIS3 knockdown significantly reduced the migratory capacity of STAD cells, whereas GLIS3 overexpression enhanced cell migration (**Figure 3D**). CCK-8 assays showed that GLIS3 significantly promoted cell proliferation (*P* < 0.001; **Figure 3E**). Consistently, colony formation assays revealed that GLIS3 knockdown markedly decreased, while GLIS3 overexpression increased, clonogenic growth (**Figure 3F**).

**Figure 4:**
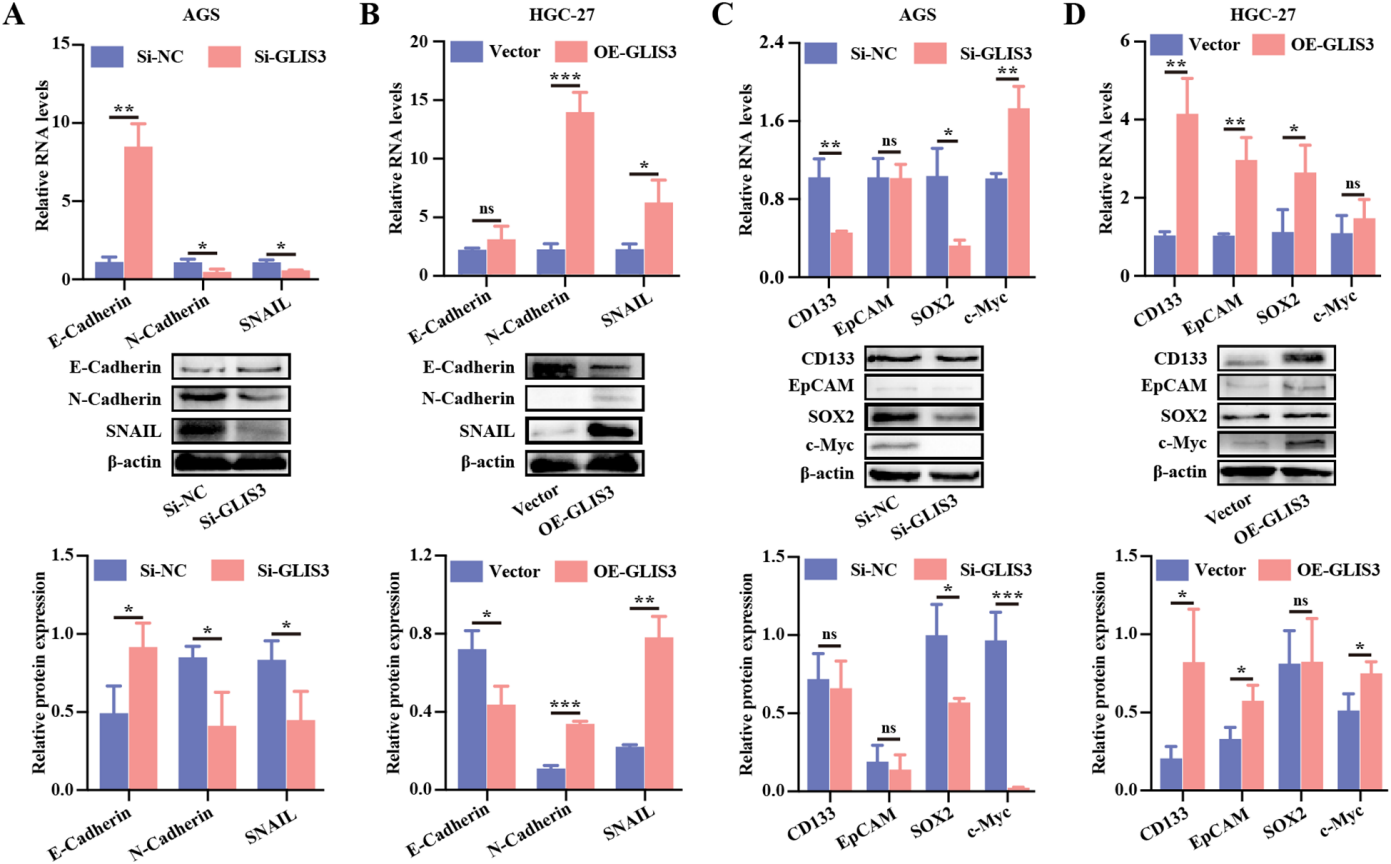
GLIS3 regulates EMT and CSC-like traits in STAD cells. **(A, B)** Effects of GLIS3 knockdown and overexpression on EMT marker expression; **(C, D)** Effects of GLIS3 knockdown and overexpression on CSC marker expression. * *P* < 0.05, ** *P* < 0.01, and *** *P* < 0.001; ns, *P* ≥ 0.05 (n = 3 independent experiments).

We next examined the impact of GLIS3 on EMT- and CSC-related molecules. In AGS cells, GLIS3 knockdown increased E-cadherin expression while reducing N-cadherin and SNAIL levels, as confirmed by RT-qPCR and WB (*P* < 0.05; **Figure 4A**). In HGC-27 cells, GLIS3 overexpression was associated with increased N-cadherin and SNAIL expression, whereas the E-cadherin signal was interpreted cautiously as a low-baseline supportive readout (**Figure 4B**). Similarly, CSC markers, including CD133, EpCAM, SOX2 and c-Myc, were downregulated upon GLIS3 knockdown and upregulated following GLIS3 overexpression (*P* < 0.05; **Figures 4C, D**). These findings indicate that GLIS3 enhances proliferation, migration, EMT and stem-like traits in STAD cells.

### GLIS3 transcriptionally activates TGFBR3 and engages Hedgehog signaling to promote EMT/CSC phenotypes

**Figure 5:**
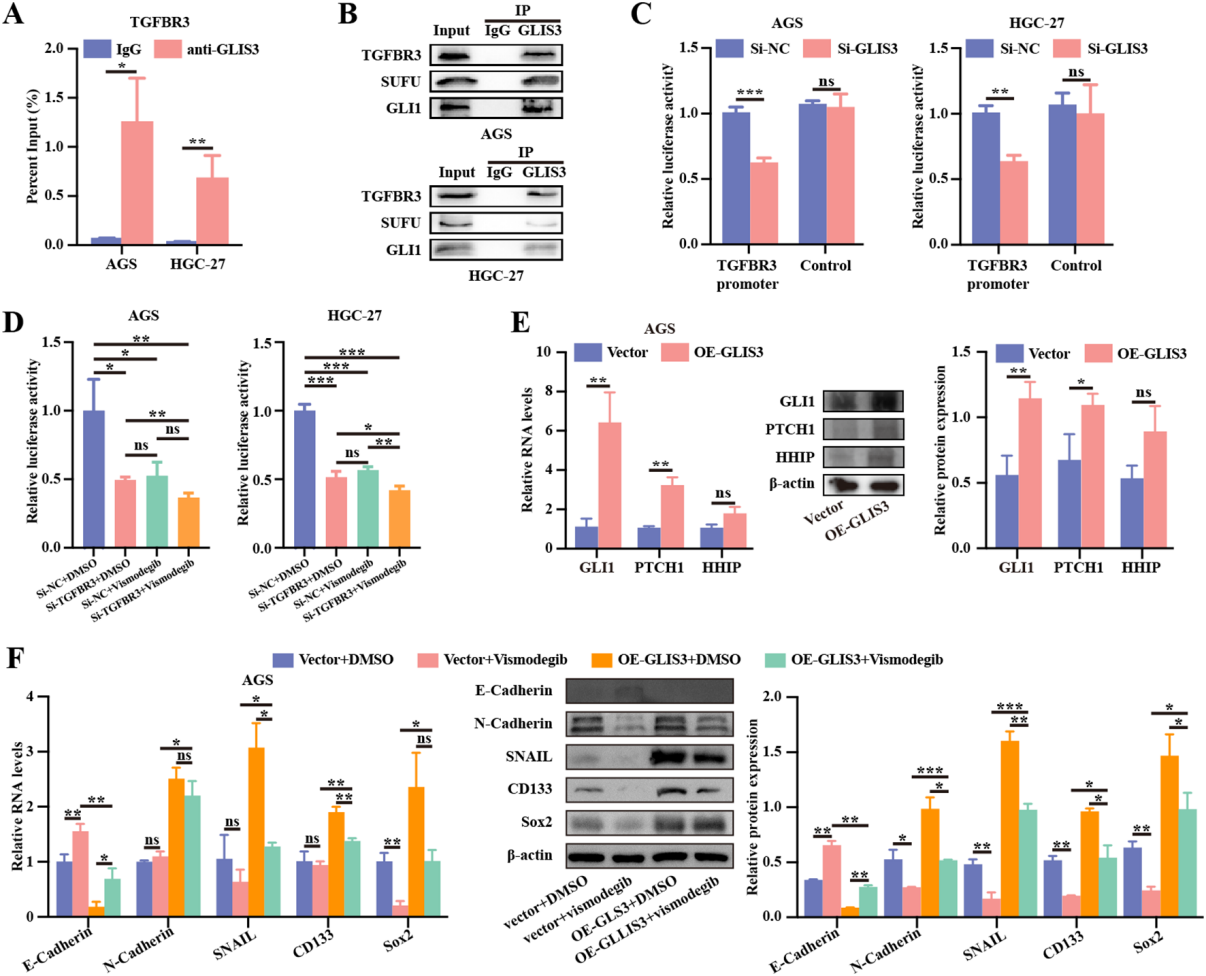
The GLIS3–TGFBR3–Hedgehog axis mediates the pro-EMT and pro-CSC effects of GLIS3 in STAD cells. **(A)** ChIP-qPCR showing significant enrichment of GLIS3 at the TGFBR3 promoter; **(B)** Co-immunoprecipitation assays using whole-cell lysates demonstrating that TGFBR3, SUFU, and GLI1 were detected in GLIS3 immunoprecipitates; **(C)** Dual-luciferase reporter assays showing reduced TGFBR3 promoter activity following GLIS3 knockdown; **(D)** 8×GLI luciferase reporter assays showing GLI-dependent transcriptional activity after TGFBR3 knockdown with or without vismodegib treatment in AGS and HGC-27 cells; **(E)** RT-qPCR and WB analyzes showing increased expression of Hedgehog pathway target genes following GLIS3 overexpression; **(F)** Effects of vismodegib on EMT- and CSC-related marker expression in AGS cells under complete control conditions (Vector + DMSO, Vector + vismodegib, OE-GLIS3 + DMSO, and OE-GLIS3 + vismodegib), as assessed by RT-qPCR and WB. * *P* < 0.05, ** *P* < 0.01, and *** *P* < 0.001; ns, *P* ≥ 0.05 (n = 3 independent experiments); ChIP-qPCR, Chromatin immunoprecipitation followed by quantitative PCR.

We next investigated whether GLIS3 drives EMT and CSC phenotypes through downstream effector pathways. Through integrative analysis of the GTRD and PWMEnrich databases combined with TCGA data, we identified 103 potential GLIS3 target genes (**Supplementary Figure 1A**). Among these candidates, TGFBR3 was prioritized because of its association with Hedgehog pathway–related signatures. ChIP assays in AGS and HGC-27 cells demonstrated significant enrichment of GLIS3 at the TGFBR3 promoter, suggesting transcriptional activation of TGFBR3 by GLIS3 (*P* < 0.05; **Figure 5A**). To assess motif dependence, two promoter mutants disrupting the putative GLIS3-binding motifs (Mut1 and Mut2), as well as a double mutant (Mut1 + 2), were generated. GLIS3 knockdown markedly reduced wild-type TGFBR3 promoter activity, whereas this effect was attenuated in the single mutants and further diminished in the double mutant in both AGS and HGC-27 cells (**Supplementary Figure 1B**). Consistent with its receptor localization, subcellular fractionation showed that endogenous TGFBR3 was predominantly detected in the membrane fraction, with minimal nuclear signal (**Supplementary Figure 1D**). Under the corresponding ChIP conditions, enrichment at the predefined TGFBR3 and GLI1 promoter amplicons was reduced after GLIS3 or TGFBR3 silencing, respectively, whereas IgG and negative-region signals remained low (**Supplementary Figures 1E, F**). Furthermore, co-IP assays using whole-cell lysates revealed that TGFBR3, SUFU, and GLI1 were detectable in GLIS3 immunoprecipitates (**Figure 5B**), supporting a biochemical association among these proteins under the tested conditions. Given their distinct subcellular distributions, this interaction may occur in membrane-proximal or cytoplasmic compartments and may modulate GLI/SUFU trafficking and transcriptional output, rather than implying that TGFBR3 itself functions as a nuclear transcription factor. In line with these findings, dual-luciferase assays confirmed that GLIS3 knockdown significantly reduced TGFBR3 promoter activity in both AGS and HGC-27 cells (*P* < 0.01; **Figure 5C**). To functionally assess GLI-dependent transcriptional output, we performed an 8×GLI luciferase reporter assay. In both AGS and HGC-27 cells, TGFBR3 silencing and vismodegib each significantly reduced GLI reporter activity, and the combined si-TGFBR3 + vismodegib condition yielded the lowest reporter activity among the tested groups (**Figure 5D**). Two-way ANOVA demonstrated significant main effects of si-TGFBR3 and vismodegib, as well as a significant interaction, in AGS cells (si-TGFBR3: F = 20.62, *P* = 0.0019; vismodegib: F = 17.19, *P* = 0.0032; interaction: F = 5.67, *P* = 0.0444) and in HGC-27 cells (si-TGFBR3: F = 215.3, *P* = 4.57 × 10^-7^; vismodegib: F = 149.87, *P* = 1.84 × 10^-6^; interaction: F = 61.72, *P* = 4.97 × 10^-5^). Consistently, in HGC-27 cells, RT-qPCR analysis of endogenous Hedgehog target genes showed that either TGFBR3 silencing or vismodegib alone reduced transcript levels to 0.5–0.6 of control, whereas the si-TGFBR3 + vismodegib group showed a trend toward the lowest mean expression (**Supplementary Figure 1G**). Consistent with earlier findings of GLIS3–Hedgehog pathway interactions, overexpression of GLIS3 increased Hedgehog pathway readouts, with the most consistent changes observed for GLI1 and PTCH1 (**Figure 5E**). In AGS cells, GLIS3 overexpression increased EMT- and CSC-associated markers, whereas vismodegib treatment partially reversed these changes, as evidenced by increased E-cadherin together with reduced N-cadherin, SNAIL, CD133, and SOX2 at the mRNA and protein levels (*P* < 0.05; **Figure 5F**).

### TGFBR3 mediates GLIS3-induced EMT and stem-like phenotypes

**Figure 6:**
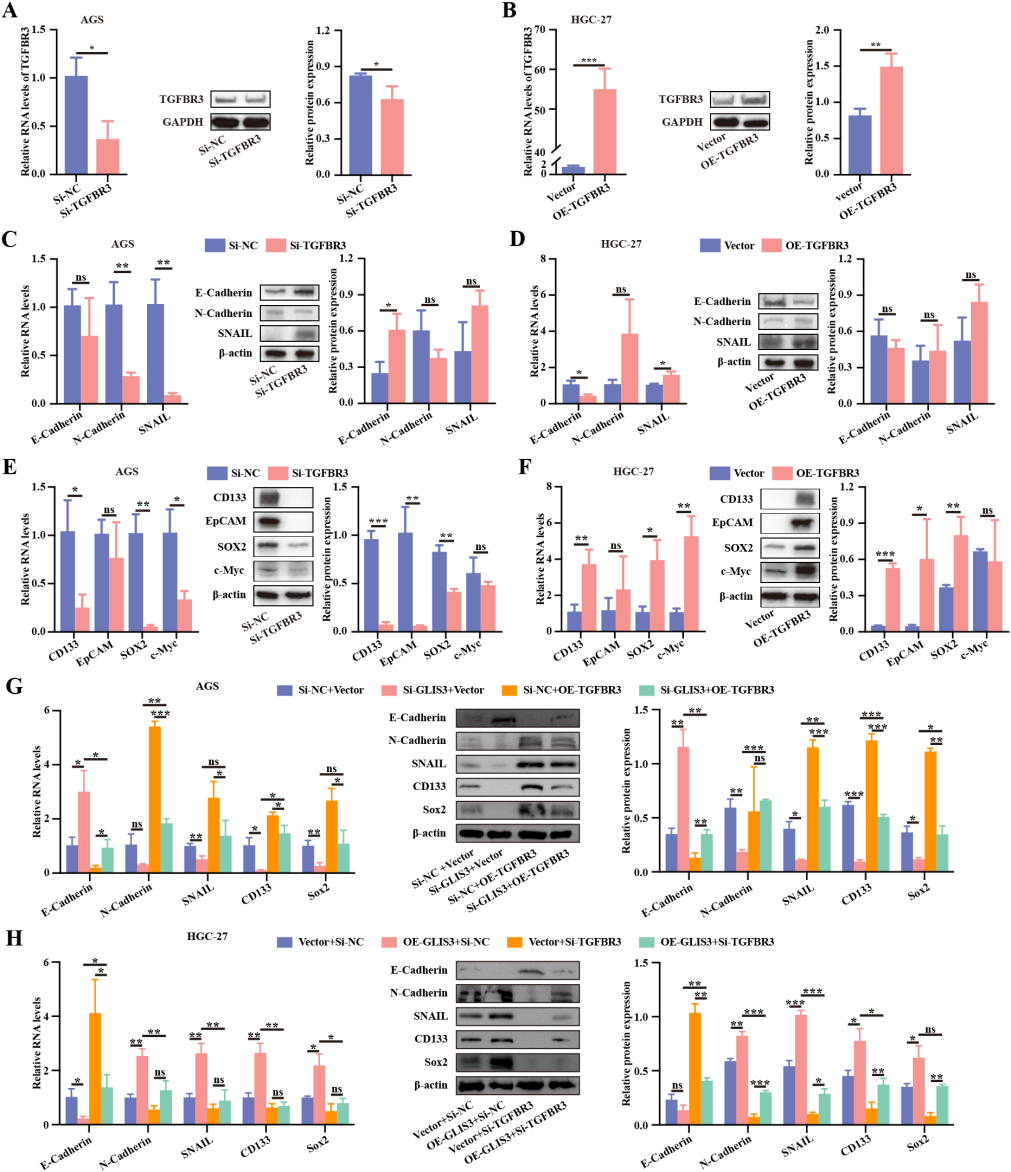
TGFBR3 mediates GLIS3-driven EMT and CSC-like traits in STAD cells. **(A)** Validation of TGFBR3 knockdown efficiency by RT-qPCR and WB; **(B)** Validation of TGFBR3 overexpression efficiency by RT-qPCR and WB (OE-TGFBR3 insert corresponds to NM_001195683.2 nt 388–2940); **(C)** Effects of TGFBR3 knockdown on EMT marker expression; **(D)** Effects of TGFBR3 overexpression on EMT marker expression; **(E)** Effects of TGFBR3 knockdown on CSC marker expression; **(F)** Effects of TGFBR3 overexpression on CSC marker expression; **(G)** Rescue of EMT- and CSC-related marker expression in AGS cells following combined GLIS3 knockdown and TGFBR3 overexpression; **(H)** Rescue of EMT- and CSC-related marker expression in HGC-27 cells following combined GLIS3 overexpression and TGFBR3 knockdown. * *P* < 0.05, ** *P* < 0.01, and *** *P* < 0.001; ns, *P* ≥ 0.05 (n = 3 independent experiments).

Having identified TGFBR3 as a downstream effector of GLIS3, we next examined whether TGFBR3 independently modulates EMT and CSC traits. RT-qPCR and WB confirmed efficient TGFBR3 knockdown and overexpression (*P* < 0.05; **Figures 6A, B**). To further evaluate siRNA specificity and protein-level suppression, two independent siRNAs targeting TGFBR3 were screened in AGS cells, and both reduced TGFBR3 expression at the mRNA and protein levels (**Supplementary Figure 3D**). In the si-TGFBR3 group, N-cadherin and SNAIL mRNA levels were significantly reduced compared with the negative-control group, whereas E-cadherin protein expression was increased (*P* < 0.05; **Figure 6C**). Conversely, TGFBR3 overexpression decreased E-cadherin and increased N-cadherin and SNAIL expression (**Figure 6D**). CSC-associated markers followed a similar pattern, with reduced expression after TGFBR3 silencing and increased expression upon TGFBR3 overexpression (*P* < 0.05; **Figures 6E, F**). Functional assays further demonstrated that TGFBR3 knockdown suppressed, whereas its overexpression enhanced, cell migration and proliferation (**Supplementary Figures 1H–J**).

To further interrogate the causal hierarchy within this axis, we performed bidirectional rescue experiments combining GLIS3 and TGFBR3 perturbations. In AGS cells, TGFBR3 overexpression partially reversed the GLIS3-knockdown-induced epithelial shift and restored stemness-associated markers, as evidenced by decreased E-cadherin and increased N-cadherin, SNAIL, CD133, and SOX2 relative to the si-GLIS3 + Vector group (**Figure 6G**). Conversely, in HGC-27 cells, TGFBR3 silencing attenuated the EMT- and stemness-promoting effects of GLIS3 overexpression, as reflected by increased E-cadherin and reduced N-cadherin, SNAIL, CD133, and SOX2 compared with the OE-GLIS3 + si-NC group (**Figure 6H**). At the functional level, TGFBR3 overexpression partially restored wound closure, proliferative capacity, and colony formation in GLIS3-silenced AGS cells, whereas TGFBR3 knockdown blunted the corresponding GLIS3-driven phenotypes in HGC-27 cells (**Supplementary Figures 2A–C**). Together, these findings support TGFBR3 as a functional mediator of GLIS3-driven EMT and CSC-like phenotypes in STAD cells.

### GLIS3 knockdown suppresses xenograft growth and reverses Hedgehog/EMT/CSC programs *in vivo*

**Figure 7:**
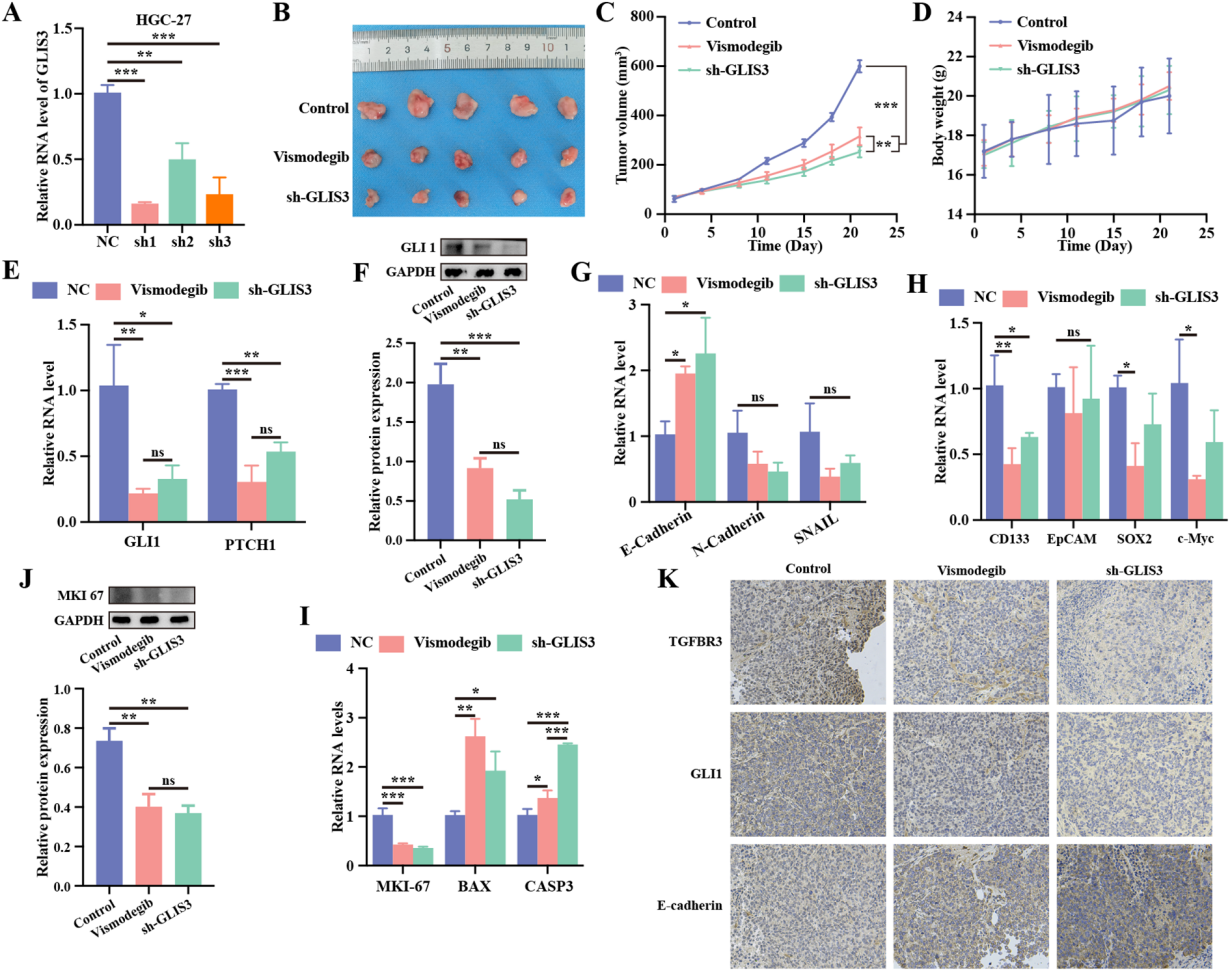
Xenograft tumor model in nude mice. **(A)** Validation of shRNA transfection efficiency; **(B)** Representative images of subcutaneous tumors in nude mice; **(C)** Tumor volume measurements over time; **(D)** Body weight monitoring of mice throughout the experiment; **(E)** RT-qPCR analysis of key Hedgehog pathway components in tumor tissues; **(F)** WB of GLI1 protein levels in tumor tissues; **(G)** RT-qPCR analysis of EMT-related gene expression; **(H)** RT-qPCR analysis of CSC marker expression; **(I)** RT-qPCR analysis of proliferation- and apoptosis-related gene expression; **(J)** WB of MKI67 protein levels in tumor tissues; **(K)** Representative immunohistochemical staining of TGFBR3, GLI1, and E-cadherin in xenograft sections from the control, vismodegib, and sh-GLIS3 groups. * *P* < 0.05, ** *P* < 0.01, and *** *P* < 0.001; ns, *P* ≥ 0.05 (n = 3 independent experiments).

To validate the *in vitro* findings, subcutaneous xenograft models were established using HGC-27 cells stably expressing GLIS3 shRNA. Tumor growth was compared with that of control xenografts and xenografts treated with the Hedgehog inhibitor vismodegib. No pain-related behaviors were observed, all mice survived to the experimental endpoint, and no unexpected deaths occurred. Transfection efficiency of three shRNA constructs was validated *in vitro* (**Figure 7A**), and the most effective construct (sh1) was selected for *in vivo* experiments. Macroscopic inspection (**Figure 7B**) and serial tumor measurements (**Figure 7C**) demonstrated that sh-GLIS3 tumors grew significantly more slowly than control tumors from day 10 onward (*P* < 0.001 on day 21), while vismodegib treatment produced intermediate but significant growth inhibition (*P* < 0.01). Although sh-GLIS3 tumors were numerically smaller than those in the vismodegib group, this observation should be interpreted as model- and regimen-dependent rather than a definitive statement of clinical superiority. Body weight changes were comparable among groups, indicating no overt systemic toxicity (**Figure 7D**).

Consistent with Hedgehog pathway suppression, GLI1 and PTCH1 transcript levels were significantly reduced in sh-GLIS3 xenografts (GLI1: *P* < 0.05; PTCH1: *P* < 0.01; **Figure 7E**), with concordant reductions in GLI1 protein expression (*P* < 0.01; **Figure 7F**). Spatially resolved IHC further demonstrated reduced TGFBR3 and GLI1 staining together with increased E-cadherin staining in sh-GLIS3 tumors; vismodegib-treated tumors likewise showed reduced GLI1 staining and stronger E-cadherin staining than controls (**Figure 7K**). To further substantiate Hedgehog pathway attenuation at the transcript level *in vivo*, additional Hedgehog targets were quantified in xenograft tissues by RT-qPCR, which revealed a concordant reduction pattern in the sh-GLIS3 group (**Supplementary Figure 3E**). GLIS3 depletion increased E-cadherin while diminishing N-cadherin and SNAIL expression (**Figure 7G**). Stemness-associated markers were likewise downregulated (**Figure 7H**), consistent with the *in vitro* findings. Given the distinct intervention levels of GLIS3 silencing and vismodegib, we next assessed whether combined perturbation could yield additional anti-proliferative effects *in vitro*. In HGC-27 cells, the combination of GLIS3 knockdown with vismodegib further reduced cell viability compared with either single intervention (**Supplementary Figure 3F**). At the pathway level, RT-qPCR of canonical Hedgehog readouts confirmed that both vismodegib and GLIS3 knockdown dampened Hedgehog transcriptional outputs, whereas the combination did not uniformly produce further suppression across all targets, consistent with convergent regulation and potential feedback effects at canonical Hedgehog endpoints (**Supplementary Figure 3G**).

Proliferation was curtailed, as evidenced by a sharp decrease in MKI67 mRNA and protein (RT-qPCR: *P* < 0.001; WB: *P* < 0.01) (**Figures 7I, J**). Concurrently, pro-apoptotic BAX and CASP3 transcripts were elevated (*P* < 0.05; **Figure 7I**), indicating the activation of apoptotic pathways. Collectively, both GLIS3 silencing and SMO inhibition with vismodegib suppressed tumor growth and attenuated Hedgehog pathway output *in vivo*, with a trend toward a larger reduction in the sh-GLIS3 arm, accompanied by reversal of EMT and CSC phenotypes.

## Discussion

By integrating transcriptomic analyzes, patient specimens, mechanistic assays, and xenograft models, our study supports the notion that GLIS3 promotes malignant progression in STAD. Across independent datasets, GLIS3 expression was consistently elevated in tumor tissues compared with matched non-tumor mucosa and was associated with greater nodal burden, more advanced nodal and metastatic status, and reduced OS, PFS, and DSS. Functionally, GLIS3 enhanced proliferation, migration, mesenchymal transition, and the acquisition of CSC-like traits in STAD cells, whereas GLIS3 silencing exerted opposing effects. Because HGC-27 is a poorly differentiated metastatic gastric cancer cell line widely regarded as E-cadherin-deficient/low, the E-cadherin signal was not interpreted as evidence that HGC-27 behaves as an E-cadherin-intact epithelial model; rather, it was treated as a weak supportive readout alongside the more informative induction of mesenchymal markers, the reciprocal AGS findings, and the concordant functional and rescue data. Mechanistically, our data indicate that GLIS3 upregulates TGFBR3, thereby modulating Hedgehog signaling, which is associated with enhanced EMT and CSC features and accelerated tumor growth. *In vivo*, stable GLIS3 knockdown markedly suppressed xenograft tumor growth and reversed Hedgehog, EMT, and CSC marker expression. Importantly, xenograft IHC provided spatially resolved evidence that sh-GLIS3 tumors exhibited reduced TGFBR3 and GLI1 staining together with restored E-cadherin expression, thereby strengthening the biological interpretation of the *in vivo* signaling relationship. These findings support an *in vivo* functional role for the GLIS3–TGFBR3–Hedgehog axis. Collectively, our results suggest that GLIS3 may serve as a key regulatory node and a potential therapeutic vulnerability in STAD.

To our knowledge, this regulatory circuit has not been previously described in STAD. GLIS3 has been implicated in other malignancies, including breast cancer, glioma, and leukemia, where it promotes proliferation, invasion, or lineage plasticity (21–23). Zhang et al. reported that, in STAD, GLIS3 facilitates proliferation and migration via the TGF-β1/Smad1/5 pathway (15). Our work extends these findings in three important ways: First, we provide comprehensive functional evidence that GLIS3 directly reinforces EMT and CSC-like phenotypes, as demonstrated by coordinated regulation of epithelial and mesenchymal markers and multiple stemness-related genes in both gain- and loss-of-function experiments. Second, we identify TGFBR3 as a direct transcriptional target of GLIS3 and position it at the interface between GLIS3 and Hedgehog signaling. ChIP and dual-luciferase assays support a model in which GLIS3 binds the TGFBR3 promoter to enhance Hedgehog pathway activity. Given its predominant membrane localization, we do not propose that TGFBR3 functions as a nuclear DNA-binding regulator in our model. Instead, our data support a membrane-proximal mechanism in which TGFBR3 modulates Hedgehog transcriptional output through regulation of the SUFU–GLI module. Importantly, whole-cell co-IP does not resolve the subcellular site of interaction. Consistent with our fractionation data, endogenous TGFBR3 is predominantly localized to membrane fractions with minimal nuclear signal, whereas SUFU and GLI1 shuttle between cytosolic and nuclear compartments (24). Thus, the co-IP data should be interpreted as evidence of a biochemical association within immunoprecipitated complexes, not as definitive proof of a stable multi-protein complex in a single compartment or a nuclear transcriptional function for TGFBR3. A plausible working model is that membrane-associated TGFBR3-containing complexes interact with the SUFU–GLI module at the membrane–cytosol interface or within membrane-associated intracellular compartments (e.g., endomembrane trafficking vesicles), thereby influencing SUFU–GLI trafficking and Hedgehog transcriptional output (25). Definitive localization of these interactions will require fractionation-resolved interaction assays or proximity-based imaging techniques. Third, we extend beyond *in vitro* studies by demonstrating that GLIS3 silencing attenuates Hedgehog signaling, reverses EMT and CSC traits, and suppresses tumor growth *in vivo*, providing biologically relevant validation of this signaling axis. Importantly, the revised bidirectional rescue experiments further show that TGFBR3 mediates, at least in part, the EMT/CSC-like and growth-related phenotypes driven by GLIS3, thereby strengthening the causal hierarchy of the proposed signaling axis.

These findings have several mechanistic and translational implications. EMT and CSC programs are tightly linked to tumor plasticity, the invasion–metastasis cascade, and resistance to chemotherapy and targeted agents in STAD (26–29). Our enrichment analyzes indicate that GLIS3-associated transcriptomes are enriched not only for EMT and CSC signatures but also for Hedgehog signaling, a known regulator of GCSC self-renewal and survival (30). Beyond Hedgehog, GLIS3-related gene sets also pointed to MAPK, Wnt, and Notch signaling, suggesting that GLIS3 may interface with broader oncogenic networks, although these axes were not explored mechanistically in the present study. Together with our functional data, this supports the concept that GLIS3 acts as an upstream coordinator of mesenchymal and stem-like states via TGFBR3-mediated activation of Hedgehog. Notably, both sh-GLIS3 and vismodegib reduced tumor burden compared with control, and the sh-GLIS3 group showed numerically smaller tumors under the tested conditions. This observation is consistent with the possibility that GLIS3 silencing may exert broader transcriptional effects in some contexts, potentially dampening multiple EMT-/CSC-supporting programs, whereas vismodegib targets a single node within canonical Hedgehog signaling. Therefore, the magnitude of response may vary with baseline pathway activation, inhibitor exposure, and intrinsic resistance mechanisms (31). These results raise the possibility that therapeutic strategies targeting GLIS3 itself, its interaction with the TGFBR3 promoter, or nodal components of the GLIS3–TGFBR3–Hedgehog axis might offer advantages over conventional pharmacologic Hedgehog pathway blockade, particularly in patients whose tumors exhibit high GLIS3 expression and prominent EMT/CSC features. Combined-targeting data indicate that, compared with either single intervention, the dual perturbation further suppressed cell proliferation and, to some extent, Hedgehog transcriptional output. Although these findings do not establish clinical benefit, they provide a mechanistic rationale for future dose–response and pharmacodynamic studies.

Clinically, responses to SMO inhibitors such as vismodegib can be heterogeneous and are frequently constrained by both intrinsic and acquired resistance. Canonical resistance commonly involves SMO alterations that impair drug binding (e.g., the D473H mutation) or reactivate Hedgehog output despite SMO blockade (32), while additional mechanisms include pathway reactivation through copy-number changes and downstream lesions such as SUFU loss and GLI2 amplification (33). These events collectively enable tumors to maintain GLI-dependent transcription even under pharmacologic SMO inhibition. Moreover, the tolerability profile of vismodegib (e.g., muscle spasms, dysgeusia, alopecia, fatigue, and weight loss) may limit drug exposure and thereby attenuate pathway suppression *in vivo (*34). Beyond tumor-intrinsic lesions, tumor–stroma interactions have also been implicated in therapeutic escape and pathway reactivation. Therefore, biomarkers that indicate Hedgehog pathway engagement may help stratify patients and guide rational combination strategies.

Some limitations of this study should be acknowledged. First, the clinical correlations were derived from a single-center cohort with a modest sample size and retrospective follow-up, and we lacked longitudinal treatment information as well as prospective, multicenter validation to establish the generalizability of GLIS3 as a prognostic biomarker. Second, our *in vivo* validation relied on subcutaneous xenografts generated from a single STAD cell line in immunodeficient nude mice; therefore, the current data do not capture orthotopic or metastatic microenvironmental features, nor do they allow direct assessment of how GLIS3 modulates antitumor immunity or interacts with systemic therapies. Third, although we delineated a GLIS3–TGFBR3–Hedgehog axis at the transcriptional and functional levels, we did not investigate upstream genomic or epigenetic events that drive GLIS3 dysregulation. Fourth, we did not generate direct evidence that GLIS3 status predicts response to chemotherapy, targeted agents, or immunotherapy, and vismodegib was evaluated using a single *in vitro* concentration and a single *in vivo* dosing regimen without systematic dose–response characterization, pharmacodynamic profiling, or resistance-event mapping. Accordingly, the comparative magnitude of effect between GLIS3 silencing and SMO inhibition should be viewed as context-dependent and hypothesis-generating rather than as a definitive assessment of clinical superiority. Future studies should validate GLIS3-centered networks in immunocompetent and clinically relevant models, define upstream regulatory mechanisms controlling GLIS3 expression, and test pharmacological or genetic strategies to inhibit GLIS3 or disrupt its regulation of TGFBR3. It will also be important to evaluate GLIS3-guided combinatorial regimens that integrate modulation of the GLIS3 axis with Hedgehog inhibitors, cytotoxic agents, and/or immunomodulatory approaches to clarify whether GLIS3 represents not only a biomarker of aggressive disease but also a tractable therapeutic vulnerability in STAD.

## Conclusion

Our findings support a model in which GLIS3 promotes STAD progression through a TGFBR3–Hedgehog program associated with EMT and CSC-like phenotypes. Beyond clarifying a mechanistic axis, these findings have potential translational relevance by supporting GLIS3-based risk stratification and providing a rationale for future biomarker-guided studies targeting the GLIS3–TGFBR3–Hedgehog axis, potentially in combination with Hedgehog pathway inhibition, in STAD.

## Data Availability

The original contributions presented in the study are included in the article/**Supplementary Material**. Further inquiries can be directed to the corresponding author.

## Supplementary material

Supplementary Figure 1 Target screening and functional assays. **(A)** Screening of downstream targets of GLIS3; **(B)** Motif-disrupting mutations attenuate the GLIS3-dependent activation of the TGFBR3 promoter; **(C)** ChIP-qPCR showing crosslinking-dependent enrichment of predefined GLI1 promoter amplicons recovered with anti-TGFBR3 under ChIP conditions; **(D)** Subcellular fractionation followed by immunoblotting showing predominant localization of TGFBR3 in the membrane fraction; **(E)** ChIP-qPCR analysis of GLIS3-associated chromatin at two TGFBR3 promoter regions (P1/P2) and a negative-control region; **(F)** ChIP-qPCR analysis of TGFBR3-associated chromatin at two GLI1 promoter regions (P1/P2) and a negative-control region; **(G)** RT-qPCR analysis of Hedgehog pathway target genes in cells treated with si-TGFBR3 and/or vismodegib; **(H–J)** Effects of TGFBR3 knockdown and overexpression on cell function. * *P* < 0.05, ** *P* < 0.01, and *** *P* < 0.001; ns, *P* ≥ 0.05 (n = 3 independent experiments).

Supplementary Figure 2 Functional rescue assays for the GLIS3–TGFBR3 axis in STAD cells. **(A)** Wound-healing assays under rescue conditions in AGS and HGC-27 cells; **(B)** CCK-8 assays assessing proliferative capacity under rescue conditions in AGS and HGC-27 cells; **(C)** Colony-formation assays evaluating clonogenic growth under rescue conditions in AGS and HGC-27 cells. * *P* < 0.05, ** *P* < 0.01, and *** *P* < 0.001; ns, *P* ≥ 0.05 (n = 3 independent experiments).

Supplementary Figure 3 Complementary validation of GLIS3/TGFBR3 perturbation and additional pathway analyses. **(A)** Validation of GLIS3 overexpression efficiency in AGS cells by RT-qPCR and WB; **(B)** Validation of GLIS3 knockdown efficiency in HGC-27 cells by RT-qPCR and WB; **(C)** Validation of knockdown efficiency for two independent siRNAs targeting GLIS3 in AGS cells by RT-qPCR and WB; **(D)** Validation of knockdown efficiency for two independent siRNAs targeting TGFBR3 in AGS cells by RT-qPCR and WB; **(E)** RT-qPCR quantification of additional Hedgehog pathway target genes in xenograft tissues from the treatment groups; **(F)** CCK-8 cell viability assays in HGC-27 cells under the indicated single and combined interventions; **(G)** RT-qPCR analysis of Hedgehog pathway transcriptional outputs in HGC-27 cells under the indicated conditions. * *P* < 0.05, ** *P* < 0.01, and *** *P* < 0.001; ns, *P* ≥ 0.05 (n = 3 independent experiments).
