# Supplementary figures and images for: GLIS3 drives epithelial–mesenchymal transition and cancer stem–like traits in stomach adenocarcinoma via TGFBR3–Hedgehog signaling
Source: Front Oncol. 2026 May 21;16:1826297. doi: 10.3389/fonc.2026.1826297 (PMC13233252; doi:10.3389/fonc.2026.1826297)

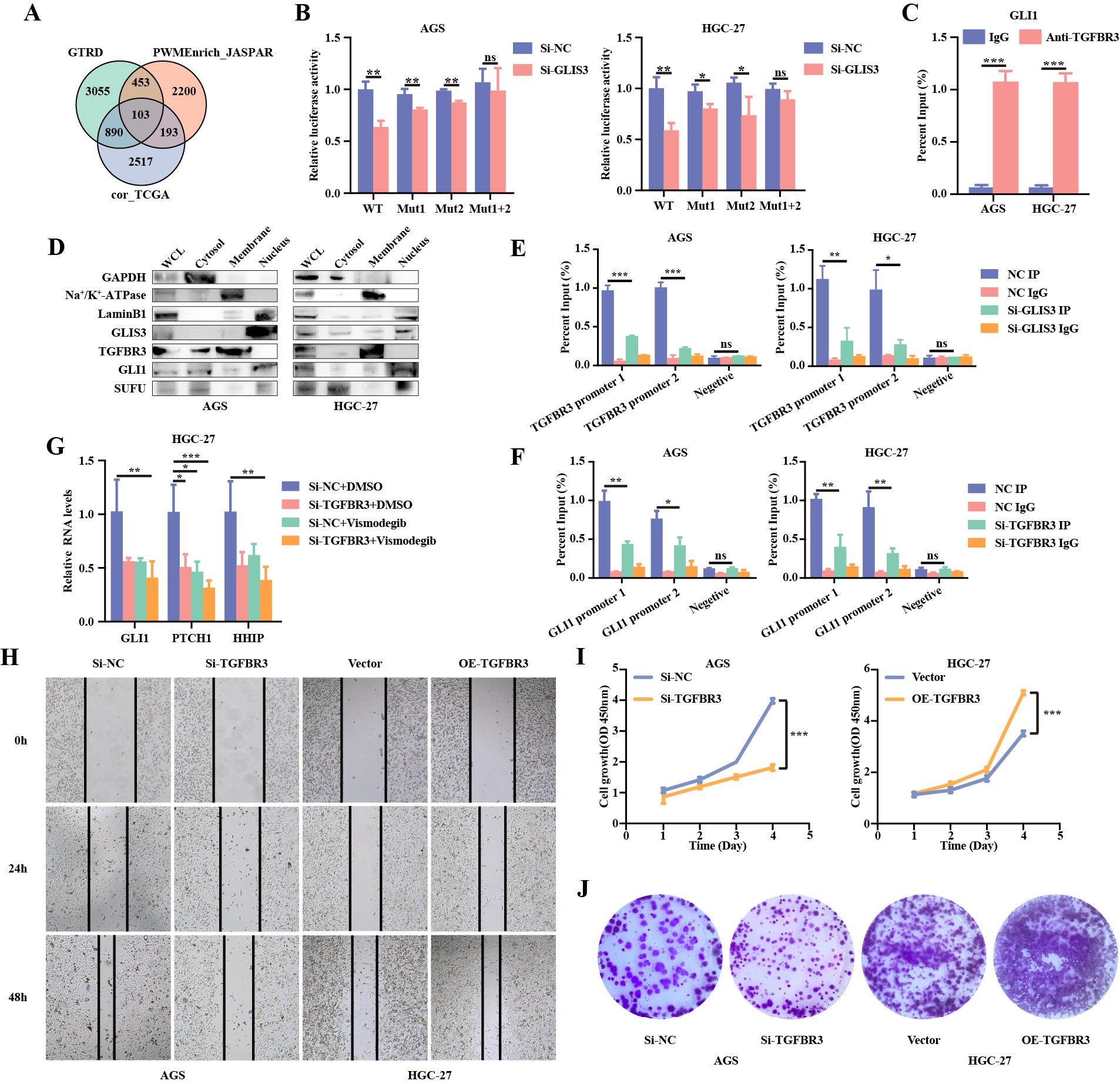

Supplement: Supplementary Figure 1 — Target screening and functional assays. (A) Screening of downstream targets of GLIS3; (B) Motif-disrupting mutations attenuate the GLIS3-dependent activation of the TGFBR3 promoter; (C) ChIP-qPCR showing crosslinking-dependent enrichment of predefined GLI1 promoter amplicons recovered with anti-TGFBR3 under ChIP conditions; (D) Subcellular fractionation followed by immunoblotting showing predominant localization of TGFBR3 in the membrane fraction; (E) ChIP-qPCR analysis of GLIS3-associated chromatin at two TGFBR3 promoter regions (P1/P2) and a negative-control region; (F) ChIP-qPCR analysis of TGFBR3-associated chromatin at two GLI1 promoter regions (P1/P2) and a negative-control region; (G) RT-qPCR analysis of Hedgehog pathway target genes in cells treated with si-TGFBR3 and/or vismodegib; (H–J) Effects of TGFBR3 knockdown and overexpression on cell function. * P < 0.05, ** P < 0.01, and *** P < 0.001; ns, P ≥ 0.05 (n = 3 independent experiments). [file Image1.tif]

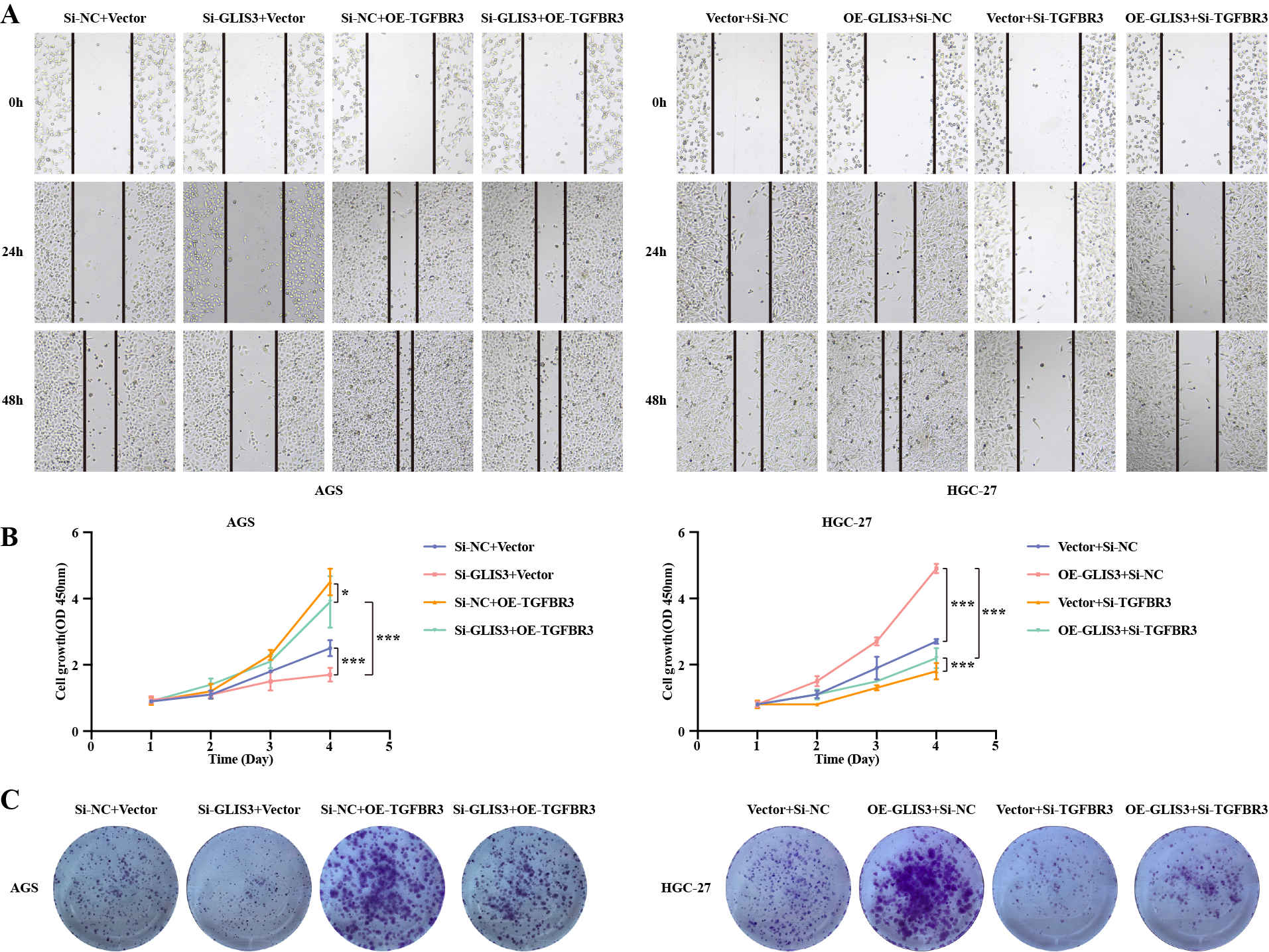

Supplement: Supplementary Figure 2 — Functional rescue assays for the GLIS3–TGFBR3 axis in STAD cells. (A) Wound-healing assays under rescue conditions in AGS and HGC-27 cells; (B) CCK-8 assays assessing proliferative capacity under rescue conditions in AGS and HGC-27 cells; (C) Colony-formation assays evaluating clonogenic growth under rescue conditions in AGS and HGC-27 cells. * P < 0.05, ** P < 0.01, and *** P < 0.001; ns, P ≥ 0.05 (n = 3 independent experiments). [file Image2.tif]

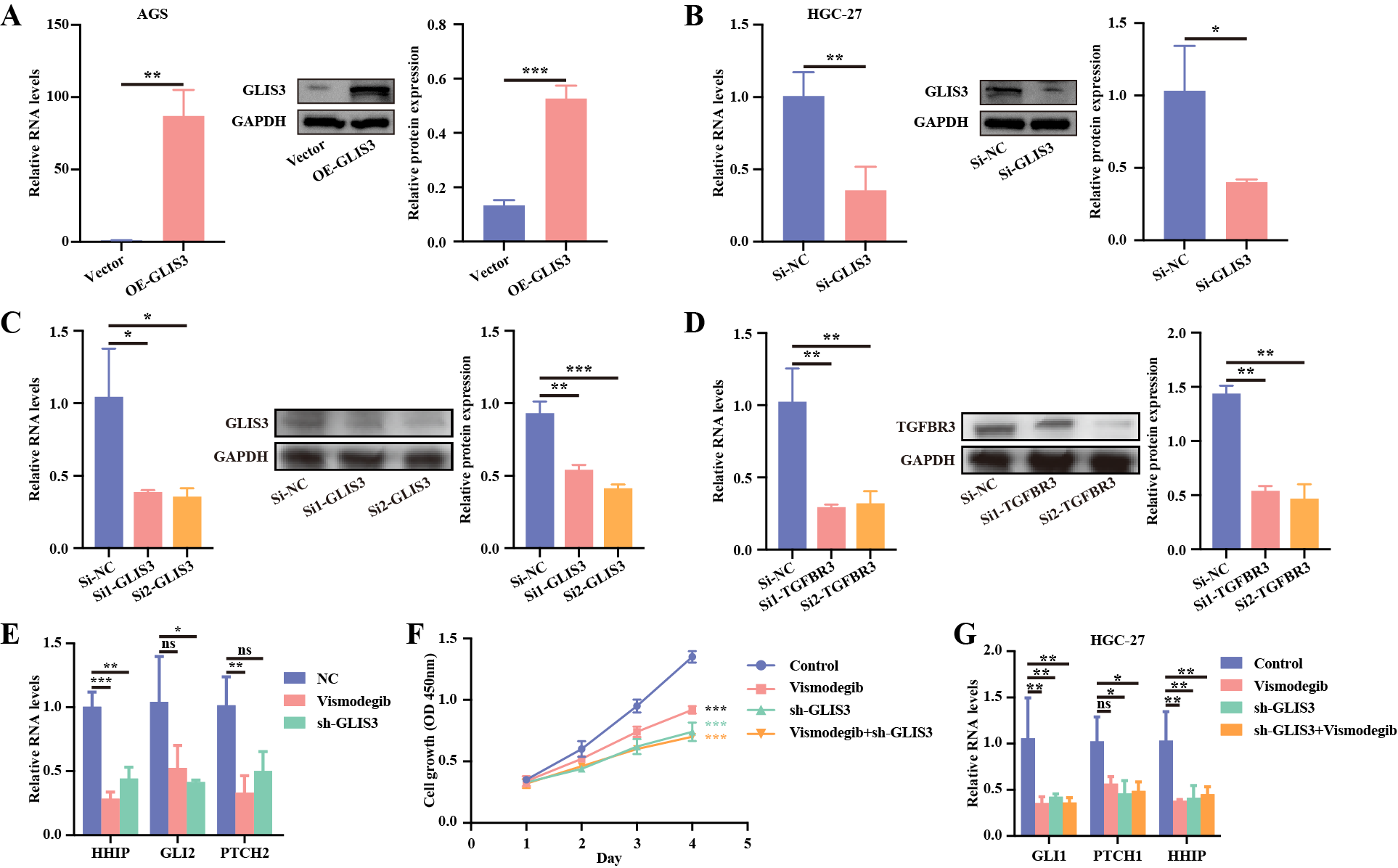

Supplement: Supplementary Figure 3 — Complementary validation of GLIS3/TGFBR3 perturbation and additional pathway analyses. (A) Validation of GLIS3 overexpression efficiency in AGS cells by RT-qPCR and WB; (B) Validation of GLIS3 knockdown efficiency in HGC-27 cells by RT-qPCR and WB; (C) Validation of knockdown efficiency for two independent siRNAs targeting GLIS3 in AGS cells by RT-qPCR and WB; (D) Validation of knockdown efficiency for two independent siRNAs targeting TGFBR3 in AGS cells by RT-qPCR and WB; (E) RT-qPCR quantification of additional Hedgehog pathway target genes in xenograft tissues from the treatment groups; (F) CCK-8 cell viability assays in HGC-27 cells under the indicated single and combined interventions; (G) RT-qPCR analysis of Hedgehog pathway transcriptional outputs in HGC-27 cells under the indicated conditions. * P < 0.05, ** P < 0.01, and *** P < 0.001; ns, P ≥ 0.05 (n = 3 independent experiments). [file Image3.tif]
