# Supplementary material for: GLIS3 drives epithelial–mesenchymal transition and cancer stem–like traits in stomach adenocarcinoma via TGFBR3–Hedgehog signaling
Source: Front Oncol. 2026 May 21;16:1826297. doi: 10.3389/fonc.2026.1826297 (PMC13233252; doi:10.3389/fonc.2026.1826297)
Supplement: Supplementary file 4 [file Table1.docx]

**Supplementary Table 1** qRT-PCR primer sequences and KD/OE sequences

| Name | Sequence |
| --- | --- |
| β-actin-F (homo) | TGGCACCCAGCACAATGAA |
| β-actin-R (homo) | CTAAGTCATAGTCCGCCTAGAAGCA |
| GLIS3-F (homo) | GTTCAGCGACTGGGACTCATT |
| GLIS3-R (homo) | CCCTCTGTAAGCTAGGACTGAT |
| E-cadherin-F (homo) | ATTTTTCCCTCGACACCCGAT |
| E-cadherin-R (homo) | TCCCAGGCGTAGACCAAGA |
| N-cadherin-F (homo) | AGCCAACCTTAACTGAGGAGT |
| N-cadherin-R (homo) | GGCAAGTTGATTGGAGGGATG |
| SNAIL-F (homo) | TGCCCTCAAGATGCACATCCGA |
| SNAIL-R (homo) | GGGACAGGAGAAGGGCTTCTC |
| CD133-F (homo) | CACTACCAAGGACAAGGCGTTC |
| CD133-R (homo) | CAACGCCTCTTTGGTCTCCTTG |
| EpCAM-F (homo) | GCCAGTGTACTTCAGTTGGTGC |
| EpCAM-R (homo) | CCCTTCAGGTTTTGCTCTTCTCC |
| SOX2-F (homo) | GCTACAGCATGATGCAGGACCA |
| SOX2-R (homo) | TCTGCGAGCTGGTCATGGAGTT |
| c-Myc-F (homo) | GTCAAGAGGCGAACACACAAC |
| c-Myc-R (homo) | TTGGACGGACAGGATGTATGC |
| TGFBR3-F (homo) | TGGGGTCTCCAGACTGTTTTT |
| TGFBR3-R (homo) | CTGCTCCATACTCTTTTCGGG |
| GLI1-F (homo) | AGCGTGAGCCTGAATCTGTG |
| GLI1-R (homo) | CAGCATGTACTGGGCTTTGAA |
| PTCH1-F (homo) | GAAGAAGGTGCTAATGTCCTGAC |
| PTCH1-R (homo) | GTCCCAGACTGTAATTTCGCC |
| HHIP-F (homo) | TCTCAAAGCCTGTTCCACTCA |
| HHIP-R (homo) | GCCTCGGCAAGTGTAAAAGAA |
| MKI67-F(homo) | ACGCCTGGTTACTATCAAAAGG |
| MKI67-R(homo) | CAGACCCATTTACTTGTGTTGGA |
| BAX-F(homo) | CCCGAGAGGTCTTTTTCCGAG |
| BAX-R(homo) | CCAGCCCATGATGGTTCTGAT |
| CASP3-F(homo) | CATGGAAGCGAATCAATGGACT |
| CASP3-R(homo) | CTGTACCAGACCGAGATGTCA |
| GLI2-F(homo) | CTGCCTCCGAGAAGCAAGAAG |
| GLI2-R(homo) | GCATGGAATGGTGGCAAGAG |
| PTCH2-F(homo) | GCTTCGTGCTTACTTCCAGGG |
| PTCH2-R(homo) | CATGCGGAGACCTAATGCCA |
| TGFBR3(ChIP)-F (homo) | GAAGAGGAAAGTGCCGCTCG |
| TGFBR3(ChIP)-R (homo) | TGATTGCTGTGGCGTCCTGC |
| TGFBR3(ChIP)-F (homo)-2 | AAATGACCGTGGACTGACAG |
| TGFBR3(ChIP)-R (homo)-2 | GCCAAGCCCTTCATTTGAAC |
| GLI1(ChIP)-F(homo)-1 | GTGGCATCGAGGCTGCGCTG |
| GLI1(ChIP)-R(homo)-1 | ATGCGCGGTCCAGGGCTGGA |
| GLI1(ChIP)-F(homo)-2 | TTCCTTGTCATGCTTCCTCCT |
| GLI1(ChIP)-R(homo)-2 | GTACTGCCTGACAGGGAAGT |
| Control(ChIP)-F(homo) | TGAGCATTCCAGTGATTTATTG |
| Control(ChIP)-R(homo) | AAGCAGGTAAAGGTCCATATTTC |
|  |  |
| Si1-GLIS3-S | GGGCAUUACAGUGUAUGAUTT |
| Si1-GLIS3-AS | AUCAUACACUGUAAUGCCCTT |
| Si2-GLIS3-S | GCAGCUGCAACAAUCUAGUTT |
| Si2-GLIS3-AS | ACUAGAUUGUUGCAGCUGCTT |
| NC-S | UUCUCCGAACGUGUCACGUTT |
| NC-AS | ACGUGACACGUUCGGAGAATT |
| OE-GLIS3 | ATGAATGGAAGATCATGCAGCATGAGTCTCCACCGGACATCGGGAACCCCACAGGGGCCTAGGATGGTCAGTGGTCATCACATTCCTGCCATCCGAGCCCACTCCGGGACTCCTGGCCCCTCGCCCTGTGGCAGCACATCGAGTCCCACTATGGCAAGCCTTGCTAACAACCTCCATCTCAAGATGCCCTCAGGAGGAGGGATGGCTCCTCAGAACAACGTGGCTGAGAGCCGCATCCATCTGCCTGCCTTAAGCCCCAGGAGACAAATGCTCACCAATGGGAAGCCGCGATTCCAGGTCACCCAGGCTGGAGGCATGTCAGGGTCACATACTTTAAAGCCAAAGCAGCAGGAGTTTGGAAGCCCTTTTCCTCCAAATCCTGGGAAAGGGGCTCTTGGCTTTGGGCCTCAGTGCAAGTCCATTGGAAAAGGCAGCTGCAACAATCTAGTGGTCACCAGCAGTCCCATGATGGTTCAGCGACTGGGACTCATTTCACCTCCAGCAAGCCAGGTCTCTACAGCATGCAACCAGATCAGTCCTAGCTTACAGAGGGCAATGAATGCAGCCAACCTGAATATACCTCCTTCAGATACCAGGTCCCTTATTTCGCGTGAGTCTTTGGCGTCCACGACCTTGAGTCTGACGGAAAGTCAGTCGGCCTCAAGCATGAAGCAGGAGTGGTCCCAGGGCTACAGGGCCCTCCCTTCGCTCTCCAACCACGGCTCTCAGAATGGCCTTGATCTAGGGGATCTCCTTAGCCTTCCTCCCGGGACATCCATGTCCAGCAATAGTGTCTCTAACTCATTACCATCCTACCTTTTTGGCACGGAAAGTAGCCACTCTCCTTACCCTAGTCCTCGGCACTCATCCACCAGGTCCCACTCGGCCCGCTCCAAGAAGAGAGCGCTGTCCTTGTCCCCGCTGTCCGATGGCATCGGGATAGATTTCAATACCATCATCCGCACGTCGCCCACGTCCTTGGTGGCCTACATCAACGGGTCGAGGGCTTCGCCGGCCAACCTGTCCCCGCAGCCGGAGGTCTACGGGCATTTCCTGGGCGTGCGCGGCAGCTGCATTCCCCAGCCGCGCCCGGTGCCCGGCAGCCAGAAGGGCGTGCTGGTGGCCCCTGGAGGCCTGGCGCTGCCGGCCTACGGCGAGGACGGGGCCCTGGAGCACGAGCGCATGCAACAGCTGGAGCACGGCGGCCTGCAGCCAGGCCTGGTCAACCACATGGTGGTGCAGCATGGCCTGCCGGGCCCCGACAGCCAGTCGGCCGGCCTGTTCAAGACCGAACGCCTGGAGGAGTTCCCGGGCAGCACCGTAGACCTACCCCCCGCGCCTCCGCTCCCTCCTCTGCCGCCGCCCCCAGGCCCCCCACCCCCTTACCATGCCCATGCGCACCTTCACCACCCGGAGCTCGGGCCCCACGCCCAGCAGCTGGCCTTGCCCCAGGCCACCCTGGACGACGACGGGGAGATGGACGGCATCGGGGGCAAGCATTGCTGCCGCTGGATCGACTGCAGCGCCCTGTACGACCAGCAGGAGGAGCTCGTGCGGCACATCGAGAAGGTCCACATCGACCAGCGCAAAGGGGAGGACTTCACTTGCTTCTGGGCCGGTTGCCCTCGAAGATACAAGCCCTTCAACGCCCGCTATAAACTGCTGATCCACATGAGAGTCCACTCTGGGGAGAAGCCCAACAAGTGTACGTTTGAAGGTTGCGAGAAGGCCTTTTCAAGGCTTGAAAATCTCAAGATCCACTTGCGGAGCCACACAGGCGAGAAGCCGTATTTGTGCCAGCATCCGGGTTGTCAGAAGGCCTTCAGTAACTCCAGTGACCGCGCCAAACACCAGCGGACGCATCTGGACACCAAACCTTATGCTTGTCAAATTCCAGGATGTACCAAACGCTACACAGACCCAAGTTCCCTAAGAAAGCATGTGAAGGCACATTCTTCCAAAGAGCAACAAGCAAGGAAAAAGTTGCGGTCCAGCACAGAGCTCCATCCAGACCTGCTCACAGATTGCCTCACCGTGCAGTCCCTGCAGCCGGCCACTTCCCCTAGAGATGCTGCTGCTGAAGGGACCGTGGGACGCTCCCCTGGACCCGGGCCTGACCTCTATTCAGCTCCCATTTTCTCCAGCAATTATTCAAGCCGAAGTGGAACAGCTGCTGGGGCCGTACCACCCCCACATCCTGTCAGTCACCCTTCTCCAGGACATAATGTACAGGGGAGCCCTCACAACCCCTCCTCCCAGTTACCTCCACTCACAGCTGTGGACGCAGGAGCTGAGAGGTTTGCACCTTCTGCTCCATCTCCTCACCACATCAGCCCCCGGAGAGTTCCAGCTCCTTCTTCAATACTGCAAAGAACACAGCCTCCCTATACCCAGCAGCCATCAGGTTCACACCTGAAGTCCTATCAGCCAGAAACAAACTCTTCTTTTCAACCAAATGGTATCCATGTCCATGGATTTTATGGGCAGCTGCAGAAGTTCTGTCCCCCACACTACCCCGATTCCCAGAGAATTGTGCCGCCTGTCAGCTCCTGCAGTGTGGTGCCTTCGTTTGAGGACTGCCTAGTCCCTACATCCATGGGCCAGGCCAGTTTTGATGTTTTCCACAGAGCCTTCTCGACTCACTCGGGCATTACAGTGTATGATTTACCTTCAAGTTCCTCGAGCCTCTTTGGGGAGTCTCTCCGCAGCGGGGCTGAAGATGCTACCTTCTTGCAGATCAGCACCGTGGACCGCTGTCCTAGCCAGCTCTCCTCTGTCTACACCGAAGGCTAA |
| Si1-TGFBR3-S | **AUCCUGUCCAGGCCUUGAU**TT |
| Si1-TGFBR3-AS | **AUCAAGGCCUGGACAGGAU**TT |
| Si2-TGFBR3-S | **AUGAAGAUCUGGAGUCAGG**TT |
| Si2-TGFBR3-AS | **CCUGACUCCAGAUCUUCAU**TT |
| NC-S | **UUCUCCGAACGUGUCACGU**TT |
| NC-AS | **ACGUGACACGUUCGGAGAA**TT |
| OE-TGFBR3 | ATGACTTCCCATTATGTGATTGCCATCTTTGCCCTGATGAGCTCCTGTTTAGCCACTGCAGGTCCAGAGCCTGGTGCACTGTGTGAACTGTCACCTGTCAGTGCCTCCCATCCTGTCCAGGCCTTGATGGAGAGCTTCACTGTTTTGTCAGGCTGTGCCAGCAGAGGCACAACTGGGCTGCCACAGGAGGTGCATGTCCTGAATCTCCGCACTGCAGGCCAGGGGCCTGGCCAGCTACAGAGAGAGGTCACACTTCACCTGAATCCCATCTCCTCAGTCCACATCCACCACAAGTCTGTTGTGTTCCTGCTCAACTCCCCACACCCCCTGGTGTGGCATCTGAAGACAGAGAGACTTGCCACTGGGGTCTCCAGACTGTTTTTGGTGTCTGAGGGTTCTGTGGTCCAGTTTTCATCAGCAAACTTCTCCTTGACAGCAGAAACAGAAGAAAGGAACTTCCCCCATGGAAATGAACATCTGTTAAATTGGGCCCGAAAAGAGTATGGAGCAGTTACTTCATTCACCGAACTCAAGATAGCAAGAAACATTTATATTAAAGTGGGGGAAGATCAAGTGTTCCCTCCAAAGTGCAACATAGGGAAGAATTTTCTCTCACTCAATTACCTTGCTGAGTACCTTCAACCCAAAGCAGCAGAAGGGTGTGTGATGTCCAGCCAGCCCCAGAATGAGGAAGTACACATCATCGAGCTAATCACCCCCAACTCTAACCCCTACAGTGCTTTCCAGGTGGATATAACAATTGATATAAGACCTTCTCAAGAGGATCTTGAAGTGGTCAAAAATCTCATCCTGATCTTGAAGTGCAAAAAGTCTGTCAACTGGGTGATCAAATCTTTTGATGTTAAGGGAAGCCTGAAAATTATTGCTCCTAACAGTATTGGCTTTGGAAAAGAGAGTGAAAGATCTATGACAATGACCAAATCAATAAGAGATGACATTCCTTCAACCCAAGGGAATCTGGTGAAGTGGGCTTTGGACAATGGCTATAGTCCAATAACTTCATACACAATGGCTCCTGTGGCTAATAGATTTCATCTTCGGCTTGAAAATAATGAGGAGATGGGAGATGAGGAAGTCCACACTATTCCTCCTGAGCTACGGATCCTGCTGGACCCTGGTGCCCTGCCTGCCCTGCAGAACCCGCCCATCCGGGGAGGGGAAGGCCAAAATGGAGGCCTTCCGTTTCCTTTCCCAGATATTTCCAGGAGAGTCTGGAATGAAGAGGGAGAAGATGGGCTCCCTCGGCCAAAGGACCCTGTCATTCCCAGCATACAACTGTTTCCTGGTCTCAGAGAGCCAGAAGAGGTGCAAGGGAGCGTGGATATTGCCCTGTCTGTCAAATGTGACAATGAGAAGATGATCGTGGCTGTAGAAAAAGATTCTTTTCAGGCCAGTGGCTACTCGGGGATGGACGTCACCCTGTTGGATCCTACCTGCAAGGCCAAGATGAATGGCACACACTTTGTTTTGGAGTCTCCTCTGAATGGCTGCGGTACTCGGCCCCGGTGGTCAGCCCTTGATGGTGTGGTCTACTATAACTCCATTGTGATACAGGTTCCAGCCCTTGGGGACAGTAGTGGTTGGCCAGATGGTTATGAAGATCTGGAGTCAGGTGATAATGGATTTCCGGGAGATATGGATGAAGGAGATGCTTCCCTGTTCACCCGACCTGAAATCGTGGTGTTTAATTGCAGCCTTCAGCAGGTGAGGAACCCCAGCAGCTTCCAGGAACAGCCCCACGGAAACATCACCTTCAACATGGAGCTATACAACACTGACCTCTTTTTGGTGCCCTCCCAGGGCGTCTTCTCTGTGCCAGAGAATGGACACGTTTATGTTGAGGTATCTGTTACTAAGGCTGAACAAGAACTGGGATTTGCCATCCAAACGTGCTTTATCTCTCCATATTCGAACCCTGATAGGATGTCTCATTACACCATTATTGAGAATATTTGTCCTAAAGATGAATCTGTGAAATTCTACAGTCCCAAGAGAGTGCACTTTCCTATCCCGCAAGCTGACATGGATAAGAAGCGATTCAGCTTTGTCTTCAAGCCTGTCTTCAACACCTCACTGCTCTTTCTACAGTGTGAGCTGACGCTGTGTACGAAGATGGAGAAGCACCCCCAGAAGTTGCCTAAGTGTGTGCCTCCTGACGAAGCCTGCACCTCGCTGGACGCCTCGATAATCTGGGCCATGATGCAGAATAAGAAGACGTTCACTAAGCCCCTTGCTGTGATCCACCATGAAGCAGAATCTAAAGAAAAAGGTCCAAGCATGAAGGAACCAAATCCAATTTCTCCACCAATTTTCCATGGTCTGGACACCCTAACCGTGATGGGCATTGCGTTTGCAGCCTTTGTGATCGGAGCACTCCTGACGGGGGCCTTGTGGTACATCTATTCTCACACAGGGGAGACAGCAGGAAGGCAGCAAGTCCCCACCTCCCCGCCAGCCTCGGAAAACAGCAGTGCTGCCCACAGCATCGGCAGCACGCAGAGCACGCCTTGCTCCAGCAGCAGCACGGCCTAG |

**1. For OE-GLIS3 and OE-TGFBR3 constructs,** the GLIS3 sequence corresponds to the human RefSeq mRNA **NM_001042413.2** and encodes the protein **NP_001035878.1**, while the TGFBR3 sequence corresponds to the human RefSeq mRNA **NM_001195683.2** and encodes the protein **NP_001182612.1**. All sequences used in this study were **identical to the corresponding coding DNA sequences (CDS)** deposited in the NCBI RefSeq database. Specifically, the GLIS3 insert corresponds to transcript nucleotides (nt) 747–3539 of NM_001042413.2, and the TGFBR3 insert corresponds to nt 388–2940 of NM_001195683.2.

2. ChIP-qPCR primers were designed and reported based on the human reference genome hg38/GRCh38. The expected amplicon sizes and genomic coordinates for each primer pair were verified and were summarized as follows: TGFBR3 **ChIP-P1: 121 bp; chr1: 91886111–91886231 (hg38); TGFBR3 ChIP-P2: 120 bp; chr1: 91887074–91887193 (hg38); GLI1 ChIP-P1: 90 bp; chr12: 57460088–57460177 (hg38); GLI1 ChIP-P2: 125 bp; chr12: 57458973–57459097 (hg38); negative control: 78 bp; chr12: 61273966–61**274043 (hg38).
