# Supplementary material for: GLIS3 drives epithelial–mesenchymal transition and cancer stem–like traits in stomach adenocarcinoma via TGFBR3–Hedgehog signaling
Source: Front Oncol. 2026 May 21;16:1826297. doi: 10.3389/fonc.2026.1826297 (PMC13233252; doi:10.3389/fonc.2026.1826297)
Supplement: Supplementary file 5 [file Table2.docx]

**Supplementary Table 2** Clinical characteristics grouped by different GLIS3 expression in TCGA - STAD cohort

| Characteristic | Low GLIS3 expression  (n = 183) | High GLIS3 expression  (n = 232) | P value |
| --- | --- | --- | --- |
| Age, median [IQR], years | 68 [61 - 74] | 66 [57 - 72] | **0.023** |
| Sex, n (%) |  |  | 0.837 |
| Male | 117 (63.9) | 151 (65.1) |  |
| Female | 66 (36.1) | 81 (34.9) |  |
| Histological grade, n (%) |  |  | 0.213 |
| G1/G2 | 79 (43.2) | 81 (34.9) |  |
| G3 | 100 (54.6) | 146 (62.9) |  |
| Unknown | 4 (2.2) | 5 (2.2) |  |
| Number of positive lymph nodes, median [IQR] | 2 [0 - 5] | 4 [0 - 10] | **<0.001** |
| Pathologic T stage, n (%) |  |  | 0.536 |
| T1/T2 | 53 (29.0) | 57 (24.6) |  |
| T3/T4 | 127 (69.4) | 169 (72.8) |  |
| Unknown | 3 (1.6) | 6 (2.6) |  |
| Pathologic N stage, n (%) |  |  | **0.047** |
| N0/N1 | 116 (63.4) | 119 (51.3) |  |
| N2/N3 | 60 (32.8) | 102 (44.0) |  |
| Unknown | 7 (3.8) | 11 (4.7) |  |
| Pathologic M stage, n (%) |  |  | **<0.001** |
| M0 | 170 (92.9) | 184 (79.3) |  |
| M1 | 8 (4.4) | 32 (13.8) |  |
| Unknown | 5 (2.7) | 16 (6.9) |  |
| AJCC pathologic stage, n (%) |  |  | **0.002** |
| I/II | 102 (55.7) | 89 (38.4) |  |
| III/IV | 72 (39.3) | 130 (56.0) |  |
| Unknown | 9 (4.9) | 13 (5.6) |  |
| Survival status, n (%) |  |  | **0.015** |
| Alive | 122 (66.7) | 127 (54.7) |  |
| Dead | 61 (33.3) | 105 (45.3) |  |

Data are presented as median (IQR) or n (%). Continuous variables were compared using the Wilcoxon rank-sum test and categorical variables using the chi-square test or Fisher’s exact test, as appropriate. All tests were two-sided. AJCC: American Joint Committee on Cancer.

Percentages for categorical variables were calculated using the full size of each GLIS3 group as the denominator, and unknown categories were retained for descriptive transparency.
