# Supplementary material for: GLIS3 drives epithelial–mesenchymal transition and cancer stem–like traits in stomach adenocarcinoma via TGFBR3–Hedgehog signaling
Source: Front Oncol. 2026 May 21;16:1826297. doi: 10.3389/fonc.2026.1826297 (PMC13233252; doi:10.3389/fonc.2026.1826297)
Supplement: Supplementary file 6 [file Table3.docx]

**Supplementary Table 3** Cox proportional hazards models evaluating the association between GLIS3 expression and overall survival in the TCGA-STAD cohort

| Variable | Category | HR (95% CI) | P value |
| --- | --- | --- | --- |
| **Univariable Cox model** |  |  |  |
| GLIS3 expression | High vs low | 1.70 (1.24 - 2.33) | **0.001** |
| Age | per 1-year increase | 1.02 (1.00 - 1.04) | **0.010** |
| Sex | Male vs female | 1.23 (0.89 - 1.71) | 0.208 |
| AJCC pathologic stage | III/IV vs I/II | 2.08 (1.49 - 2.90) | **<0.001** |
| Pathologic T stage | T3/T4 vs T1/T2 | 2.05 (1.37 - 3.06) | **<0.001** |
| Pathologic N stage | N2/N3 vs N0/N1 | 1.77 (1.29 - 2.42) | **<0.001** |
| Pathologic M stage | M1 vs M0 | 1.99 (1.27 - 3.14) | **0.003** |
| **Primary multivariable model** |  |  |  |
| GLIS3 expression | High vs low | 1.45 (1.04 - 2.02) | **0.031** |
| Age | per 1-year increase | 1.03 (1.01 - 1.04) | **0.001** |
| Sex | Male vs female | 1.09 (0.77 - 1.54) | 0.620 |
| AJCC pathologic stage | III/IV vs I/II | 2.05 (1.46 - 2.88) | **<0.001** |
| **Sensitivity multivariable model** |  |  |  |
| GLIS3 expression | High vs low | 1.41 (1.01 - 1.97) | **0.044** |
| Age | per 1-year increase | 1.03 (1.01 - 1.05) | **<0.001** |
| Sex | Male vs female | 1.08 (0.76 - 1.52) | 0.671 |
| four-level AJCC pathologic stage | II vs I | 1.50 (0.83 - 2.74) | 0.182 |
|  | III vs I | 2.50 (1.43 - 4.39) | **0.001** |
|  | IV vs I | 4.20 (2.12 - 8.31) | **<0.001** |

The primary multivariable model included GLIS3 expression, age, sex, and AJCC pathologic stage group (I/II vs III/IV).

Sensitivity multivariable model used four-level AJCC stage (I as the reference).

Cases with undefined or discrepant stage classifications were retained in Supplementary Table 2 for descriptive transparency but were excluded from Cox models requiring that variable.

Pathologic M stage was evaluated in univariable analysis but was not entered into multivariable models containing AJCC pathologic stage because M status is a component of AJCC stage.
