# Supplementary material for: GLIS3 drives epithelial–mesenchymal transition and cancer stem–like traits in stomach adenocarcinoma via TGFBR3–Hedgehog signaling
Source: Front Oncol. 2026 May 21;16:1826297. doi: 10.3389/fonc.2026.1826297 (PMC13233252; doi:10.3389/fonc.2026.1826297)
Supplement: Supplementary file 7 [file Table4.docx]

**Supplementary Table 4** Clinical characteristics grouped by different GLIS3 expression in the local STAD cohort

| Characteristic | Low GLIS3 expression (n = 65) | High GLIS3 expression (n = 68) | P value |
| --- | --- | --- | --- |
| Age, median [IQR], years | 67 [62 - 73] | 67 [62 - 73] | 0.670 |
| Sex, n (%) |  |  | 0.168 |
| Male | 55 (84.6) | 51 (75.0) |  |
| Female | 10 (15.4) | 17 (25.0) |  |
| Clinical AJCC stage at diagnosis, n (%) |  |  | **0.008** |
| I | 9 (13.8) | 8 (11.8) |  |
| II | 28 (43.1) | 12 (17.6) |  |
| III | 15 (23.1) | 24 (35.3) |  |
| IV | 13 (20.0) | 24 (35.3) |  |
| Pathologic T stage, n (%) |  |  | **0.008** |
| T1/T2 | 14 (21.5) | 4 (5.9) |  |
| T3/T4 | 51 (78.5) | 64 (94.1) |  |
| Pathologic N stage, n (%) |  |  | **<0.001** |
| N0/N1 | 53 (81.5) | 17 (25.0) |  |
| N2/N3 | 12 (18.5) | 51 (75.0) |  |
| Curative-intent surgery, n (%) |  |  | 0.079 |
| Yes | 51 (78.5) | 44 (64.7) |  |
| No | 14 (21.5) | 24 (35.3) |  |
| Neoadjuvant therapy before sampling, n (%) |  |  | 0.057 |
| Yes | 21 (32.3) | 33 (48.5) |  |
| No | 44 (67.7) | 35 (51.5) |  |
| Metastatic lesion sampling, n (%) |  |  | 0.169 |
| Yes | 4 (6.2) | 9 (13.2) |  |
| No | 61 (93.8) | 59 (86.8) |  |
| Gastrectomy type, n (%) |  |  | 0.997 |
| Total gastrectomy | 35 (53.8) | 38 (55.9) |  |
| Subtotal gastrectomy | 12 (18.5) | 12 (17.6) |  |
| Distal gastrectomy | 10 (15.4) | 10 (14.7) |  |
| Other procedures | 8 (12.3) | 8 (11.8) |  |

Data are presented as median (IQR) or n (%). Continuous variables were compared using the Wilcoxon rank-sum test and categorical variables using the chi-square test or Fisher’s exact test, as appropriate. All tests were two-sided.
