# Supplementary material for: GLIS3 drives epithelial–mesenchymal transition and cancer stem–like traits in stomach adenocarcinoma via TGFBR3–Hedgehog signaling
Source: Front Oncol. 2026 May 21;16:1826297. doi: 10.3389/fonc.2026.1826297 (PMC13233252; doi:10.3389/fonc.2026.1826297)
Supplement: Supplementary file 8 [file Table5.docx]

**Supplementary Table 5** Univariable and multivariable Weibull accelerated failure time analyses of overall survival in the curative-intent resection, primary-tumor subset of the local STAD cohort

| Variable | Category | Univariable analysis | |  | Primary multivariable model | |  | Sensitivity multivariable model | |
| --- | --- | --- | --- | --- | --- | --- | --- | --- | --- |
|  |  | TR (95% CI) | P value |  | TR (95% CI) | P value |  | TR (95% CI) | P value |
| GLIS3 expression | High vs low | 0.14 (0.06-0.34) | **<0.001** |  | 0.16 (0.07-0.38) | **<0.001** |  | 0.12 (0.04-0.34) | **<0.001** |
| Age | per 1-year increase | 1.04 (0.99-1.09) | 0.103 |  | — | — |  | — | — |
| Sex | Female vs male | 0.52 (0.22-1.25) | 0.146 |  | — | — |  | — | — |
| Clinical AJCC stage at diagnosis | III vs I/II | 0.38 (0.17-0.86) | **0.019** |  | 0.57 (0.28-1.16) | 0.122 |  | — | — |
| Pathologic T stage | T3/T4 vs T1/T2 | 0.30 (0.08-1.17) | 0.082 |  | — | — |  | — | — |
| Pathologic N stage | N2/N3 vs N0/N1 | 0.35 (0.16-0.76) | **0.009** |  | — | — |  | 1.24 (0.54-2.88) | 0.614 |
| Neoadjuvant therapy before sampling | Yes vs No | 0.34 (0.15-0.78) | **0.010** |  | — | — |  | — | — |

TRs and 95% CIs were estimated using Weibull accelerated failure time models for overall survival. A TR < 1 indicates shorter survival, whereas a TR > 1 indicates longer survival. TR: time ratio.

Clinical AJCC stage at diagnosis and pathologic N stage were evaluated in separate multivariable models rather than in the same model to avoid within-model collinearity among overlapping stage-related variables. No stage IV cases were retained in the curative-intent resection, primary-tumor subset; therefore, clinical AJCC stage at diagnosis was modeled as stage III versus stages I/II.

Analyses were restricted to patients who underwent curative-intent resection with primary-tumor samples. Complete-case dataset: n = 95, events = 42. The primary multivariable model included GLIS3 expression and clinical AJCC stage at diagnosis, whereas the sensitivity model included GLIS3 expression and pathologic N stage. Neoadjuvant therapy was included only in the univariable analysis. Clinical stage IV and pathologic M-positive cases were not present in this restricted subset.
